# Supplementary material for: Polynomial-time quantum Gibbs sampling for the weak and strong coupling regime of the Fermi-Hubbard model at any temperature
Source: Nat Commun. 2025 Nov 28;16:10736. doi: 10.1038/s41467-025-65765-1 (PMC12663229; doi:10.1038/s41467-025-65765-1)
Supplement: Supplementary file 1 — Supplementary Information [file 41467_2025_65765_MOESM1_ESM.pdf]

Supplementary Information:

Polynomial-Time Quantum Gibbs Sampling for the Weak and Strong Coupling Regime of the Fermi-Hubbard Model at any Temperature

Appendix A: Background

1. Quantum Gibbs Sampling

We start by reviewing latest literature on quantum Gibbs sampling and framework mostly developed in [3, 22]. Quantum Gibbs sampling is the task of preparing the thermal state $\sigma_\beta = e^{-\beta H}/Z$ for a quantum Hamiltonian H. This task is the quantum analogue of classical Gibbs sampling and we briefly introduce that first. Classical Gibbs sampling aims at sampling from a classical distribution $\pi(i) = e^{-\beta E(i)}/Z$ where $E(i)$ is the energy of the configuration $i \in \Omega$ of the classical system. As usual, we assume that $E(i)$ is known explicitly, but computing the partition function Z is in general intractable. The workhorse of classical Gibbs sampling is Markov chain Monte Carlo. To sample from π we construct the transition operator of a Markov chain P_{ij} which gives the probability of transitioning from i to j such that $\lim_{t\to\infty}(P^T)^t(\pi_0) = \pi$ for an arbitrary initial distribution π_0. π is called the stationary distribution of the Markov chain. A sufficient condition for π to be a stationary distribution of P is the classical detailed balance condition, which is defined as P being self-adjoint with respect to the inner product $\langle f, g\rangle_\pi = \sum_{i\in\Omega} \bar{f}_i g_i \pi_i$, where f, g are functions on the configuration space. Explicitly,

$$\langle f, Pg\rangle_\pi = \sum_{i,j\in\Omega} \bar{f}_i P_{ij} g_j \pi_i = \langle Pf, g\rangle_\pi = \sum_{i,j\in\Omega} P_{ji} \bar{f}_i g_j \pi_j$$

which is true for all f, g if

$$P_{ij}\pi_i = P_{ji}\pi_j$$

for all $i, j \in \Omega$. By summing this equation over i and using that $\sum_{i\in\Omega} P_{ji} = 1$ we indeed have $\pi P = \pi$ so that the distribution π is an eigenvector of P with eigenvalue 1. If P is also aperiodic and irreducible, then the Perron-Frobenius theorem guarantees that π is the unique stationary state of P as desired. Here, aperiodic means that the greatest common divisor of the number of transitions by which any $i \in \Omega$ can be reached starting from i is 1 and irreducible means that P has no non-trivial invariant subspaces.

In the quantum case, we can proceed analogously. We first define the quantum Markov semigroup $\mathcal{P}_t$ as the semigroup of completely positive, unital maps. While in the classical case we used discrete time dynamics for simplicity, in the quantum case it is more useful to use continuous-time dynamics, so that we can work with the generator of the dynamics called the Lindbladian $\mathcal{L}$: $\mathcal{P}_t = e^{t\mathcal{L}}$. To perform quantum Gibbs sampling, we construct a quantum Markov semigroup such that $\lim_{t\to\infty} \mathcal{P}_t^\dagger(\rho_0) = \sigma_\beta$ where ρ_0 is an arbitrary initial state and $\Phi^\dagger$ for a superoperator Φ is the adjoint w.r.t. the Hilbert-Schmidt inner product: $\langle A, B\rangle = \mathrm{Tr}(A^\dagger B)$. There are many scalar products that reduce to the classical inner product $\langle\cdot,\cdot\rangle_\pi$ when σ_β is a diagonal matrix with diagonal π. The most useful one for our purposes is the Kubo-Martin-Schwinger (KMS) inner product. Given a full rank state $\sigma > 0$, this is defined for two operators A, B as

$$\langle A, B\rangle_\sigma = \mathrm{Tr}\big(A^\dagger \Gamma_\sigma(B)\big)\,,$$

where

$$\Gamma_\sigma(A) = \sigma^{1/2} A \sigma^{1/2}\,.$$

Definition A.1. *(Quantum Detailed Balance) A Lindbladian $\mathcal{L}$ satisfies the KMS quantum detailed balance (QDB) condition if $\mathcal{L}$ is self-adjoint with respect to the KMS inner product.*

Since $\mathcal{L}[\mathbf{1}] = 0$, we have that if $\mathcal{L}$ satisfies QDB,

$$0 = \langle A, \mathcal{L}[\mathbf{1}]\rangle_\sigma = \langle \mathcal{L}[A], \mathbf{1}\rangle_\sigma = \langle \mathcal{L}[A], \sigma\rangle = \langle A, \mathcal{L}^\dagger[\sigma]\rangle\,,$$

for any operator A. This shows that $\mathcal{L}^\dagger[\sigma] = 0$ so that σ is a stationary state of the dynamics generated by $\mathcal{L}^\dagger$. We can write the QDB condition more explicitly as:

$$\mathcal{L} = \Gamma_\sigma^{-1} \circ \mathcal{L}^\dagger \circ \Gamma_\sigma\,.$$

Note that in general $\mathcal{L}$ is a non-Hermitian operator, however the self-adjointness with the KMS inner product guarantees real spectrum. We can define a Hermitian operator by a similarity transformation as follows:

Definition A.2. *(Parent Hamiltonian) Given a Lindbladian $\mathcal{L}$ satisfying QDB, we define the parent Hamiltonian of the state $\sqrt{\sigma}$, also known as the discriminant associated with the Lindbladian, as*

$$\mathcal{H} = \Gamma_\sigma^{-1/2} \circ \mathcal{L}^\dagger \circ \Gamma_\sigma^{+1/2} .$$

Hermiticity of $\mathcal{H}$ follows from the QDB condition:

$$\mathcal{H}^\dagger = \Gamma_\sigma^{+1/2} \circ \mathcal{L} \circ \Gamma_\sigma^{-1/2} = \Gamma_\sigma^{-1/2} \circ \mathcal{L}^\dagger \circ \Gamma_\sigma^{+1/2} = \mathcal{H} .$$

Also $\mathcal{H}$ has the same spectrum as $\mathcal{L}$ since they are related by a similarity transformation. In particular

$$\mathcal{H}[\sqrt{\sigma}] = \Gamma_\sigma^{-1/2} \circ \mathcal{L}^\dagger[\sigma^{1/4}\sqrt{\sigma}\sigma^{1/4}] = 0$$

which shows that indeed $\mathcal{H}$ is the parent Hamiltonian of $\sqrt{\sigma}$. Here we slightly abuse the notion of parent Hamiltonian, since $\sqrt{\sigma}$ is the highest energy state of $\mathcal{H}$ rather than the lowest one.

The Lindbladian can be written in the following form

$$\mathcal{L}^\dagger[\rho] = -i[G,\rho] + \sum_{a\in\mathcal{A}} \left(L_a \rho L_a^\dagger - \frac{1}{2}\{L_a^\dagger L_a, \rho\} \right) .$$

L_a are called the Lindblad operators and $G = G^\dagger$ is the coherent term. In terms of these operators the parent Hamiltonian is

$$\begin{aligned} \mathcal{H}[\rho] &= -i(\tilde{G}\rho - \rho\tilde{G}^\dagger) + \sum_{a\in\mathcal{A}} \left(\tilde{L}_a \rho \tilde{L}_a^\dagger - \frac{1}{2}(\tilde{M}_a\rho + \rho\tilde{M}_a^\dagger) \right) \\ \tilde{G} &= \sigma_\beta^{-1/4} G \sigma_\beta^{+1/4} , \quad \tilde{L}_a = \sigma_\beta^{-1/4} L_a \sigma_\beta^{+1/4} , \quad \tilde{M}_a = \sigma_\beta^{-1/4} L_a^\dagger L_a \sigma_\beta^{+1/4} . \end{aligned} \tag{A1}$$

Similarly to the classical case, we define irreducibility of the quantum channel $\mathcal{E}$ as the absence of invariant subspaces. More precisely if $\mathcal{E}$ acts on the space of $d \times d$ matrices $\mathcal{M}_d$, $\mathcal{E}$ is irreducible if for a projector P, the identity $\mathcal{E}(P\mathcal{M}_d P) \subseteq P\mathcal{M}_d P$ occurs only for the trivial cases of $P = 0, \mathbf{1}$ [64, Thm 6.2]. Then if $e^{t\mathcal{L}^\dagger}$ is irreducible, the kernel of $\mathcal{L}^\dagger$ is one-dimensional and spanned by the full rank density matrix σ_β [64, Prop 7.5]. This guarantees uniqueness of the stationary state. A useful criterion for irreducibility is that the algebra generated by L_a's and G is the whole operator algebra [64, Cor 7.2].

The efficiency of the Lindbladian dynamics to prepare a thermal state is governed by the mixing time.

Definition A.3. *The mixing time of the Lindbladian $\mathcal{L}^\dagger$ is*

$$t_{\mathrm{mix}}(\epsilon) = \inf \left\{ t \geq 0 \,\middle|\, \forall \rho : \left\| e^{t\mathcal{L}^\dagger}[\rho] - \sigma_\beta \right\|_{\mathrm{Tr}} \leq \epsilon \right\} ,$$

where $\|A\|_{\mathrm{Tr}} = \mathrm{Tr}\left(\sqrt{A^\dagger A}\right)$ denotes the trace norm.

Definition A.4. *A mixing time scaling polynomially in the system size n will be referred to as fast, which can be equivalently characterised by the following contractivity of the Lindbladian:*

$$\|e^{t\mathcal{L}^\dagger}[\rho] - \sigma\|_{\mathrm{Tr}} \leq \exp(\mathrm{poly}(n)) e^{-t/\,\mathrm{poly}(n)} .$$

A mixing time scaling poly-logarithmically will be referred to as rapid, which can be equivalently characterised by

$$\|e^{t\mathcal{L}^\dagger}[\rho] - \sigma\|_{\mathrm{Tr}} \leq \mathrm{poly}(n) e^{-\alpha t}$$

for some constant $\alpha > 0$.

The Lindbladian time evolution implemented on a quantum computer will prepare the following state:

Definition A.5. *The purified Gibbs state, also known as the thermofield double state, is*

$$|\sqrt{\sigma_\beta}\rangle = \frac{1}{\sqrt{\mathrm{Tr}(e^{-\beta H})}} \sum_i e^{-\beta E_i/2} |E_i\rangle|\overline{E_i}\rangle ,$$

where we follow the vectorisation convention $|\psi\rangle\langle\phi| \to |\psi\rangle|\overline{\phi}\rangle$.

This state is a vector in the doubled Hilbert space. The Gibbs state σ_β can be recovered by computing the reduced density matrix on one of the two copies of the Hilbert space. However, access to the purified Gibbs state can be more useful, allowing, for example, more efficient estimation of observables [65].

2. Construction of Lindbladians with Quantum Detailed Balance and Their Properties

Next, we review the construction of a Lindbladian that satisfies QDB for $\sigma = \sigma_\beta$. We follow the construction of [22]—the main difference from the construction of [3] is that it allows one to use a finite number of Lindblad operators. The construction is given in terms of a set of self-adjoint operators $\{A_a\}_{a\in\mathcal{A}}$ called jump operators and filter functions $\{\hat{f}^a(\nu)\}_{a\in\mathcal{A}}$ obeying

$$\hat{f}^a(\nu) = q^a(\nu)e^{-\beta\nu/4}, \quad q^a(-\nu) = \overline{q^a(\nu)}\,. \tag{A2}$$

The Lindblad operators are then given by

$$\begin{aligned} L_a &= \hat{f}^a(\mathrm{ad}_H)A^a \\ &= \sum_{\nu\in B_H} \hat{f}^a(\nu)A^a_\nu \\ &= \int_{-\infty}^{\infty} f^a(t)e^{iHt}A^a e^{-iHt}\,\mathrm{d}t \end{aligned} \tag{A3}$$

where $B_H = \{\nu = E_i - E_j\}$ is the set of Bohr frequencies, with E_i, E_j running over the spectrum of H, and

$$A_\nu = \sum_{i,j|E_i-E_j=\nu} P_i A P_j\,, \quad A = \sum_{\nu\in B_H} A_\nu\,, \quad A_\nu^\dagger = A_{-\nu}\,,$$

with P_i the projector onto the eigenspace of eigenvalue E_i. Here, $\mathrm{ad}_H X = [H, X]$ represents the adjoint endomorphism. Note that $\mathrm{ad}_H A_\nu = [H, A_\nu] = \nu A_\nu$. $\hat{f}^a(\nu)$ is the Fourier transform of $f^a(t)$. The coherent term is given by

$$\begin{aligned} G &= -i\tanh\circ\log\left(\Delta_{\sigma_\beta}^{1/4}\right)\left(\frac{1}{2}\sum_{a\in\mathcal{A}} L_a^\dagger L_a\right) \\ &= \frac{i}{2}\sum_{a\in\mathcal{A}}\sum_{\nu\in B_H}\tanh\left(\frac{\beta\nu}{4}\right)(L_a^\dagger L_a)_\nu \\ &= \sum_{a\in\mathcal{A}}\int_{-\infty}^{\infty} g(t)e^{iHt}(L_a^\dagger L_a)e^{-iHt}\,\mathrm{d}t \end{aligned} \tag{A4}$$

with

$$\hat{g}(\nu) = \frac{i}{2}\tanh\left(\frac{\beta\nu}{4}\right)\cdot\kappa(\nu)\,,$$

where $\Delta_\rho[X] = \rho X\rho^{-1}$ is the modular superoperator, and $\kappa(\nu)$ is a sort of smooth indicator function, obeying $\kappa(\nu) = 1$ on $\nu \in [-2\|H\|, 2\|H\|]$, and decaying smoothly and rapidly afterwards, so that it belongs to the class of Gevrey functions as per [22, Equation (3.17)]. In [22], it was proven that the Lindbladian so defined satisfies QDB with the thermal state σ_β.

The operators entering the parent Hamiltonian of equation (A1) then become

$$\tilde{L}_a = e^{+\beta/4H}\sum_{\nu\in B_H}\hat{f}^a(\nu)A^a_\nu e^{-\beta/4H} = \sum_{\nu\in B_H}\hat{f}^a(\nu)e^{\beta/4\nu}A^a_\nu = \int_{-\infty}^{+\infty} f^a(t+i\beta/4)e^{iHt}A^a e^{-iHt}\,\mathrm{d}t\,, \tag{A5}$$

where we used $[H, A_\nu] = \nu A_\nu$, and similarly,

$$\tilde{G} = e^{+\beta/4H}\frac{i}{2}\sum_{a\in\mathcal{A}}\sum_{\nu\in B_H}\tanh\left(\frac{\beta\nu}{4}\right)(L_a^\dagger L_a)_\nu e^{-\beta/4H} = \sum_{a\in\mathcal{A}}\int_{-\infty}^{\infty} g(t+i\beta/4)e^{iHt}(L_a^\dagger L_a)e^{-iHt}\,\mathrm{d}t\,. \tag{A6}$$

Note that $\tilde{L}_a = \tilde{L}_a^\dagger$ as $\overline{q^a(\nu)} = q^a(-\nu)$, $A_\nu^\dagger = A_{-\nu}$ and B_H is symmetric under negation. See also Lemma D.2 for the derivation of $\tilde{L}, \tilde{G}$ from a simple change of contour argument. Finally, we note that the parent Hamiltonian is frustration-free, namely $\mathcal{H} = \sum_{a\in\mathcal{A}}\mathcal{H}_a$ where each $\mathcal{H}_a$ annihilates after vectorisation the purified Gibbs state. We defer to Section A 4 a discussion on locality of the parent Hamiltonian.

A popular filter function that we will mostly focus on below is the Gaussian one

$$\hat{f}(\nu) = e^{-(\beta\nu+1)^2/8+1/8}\,, \quad f(t) = \frac{1}{2\pi}\int_{-\infty}^{\infty}\hat{f}(\nu)e^{-i\nu t}\,\mathrm{d}\nu = \sqrt{\frac{2}{\pi\beta^2}}\exp\left(-\tfrac{2}{\beta^2}\left(t - i\tfrac{\beta}{4}\right)^2\right)\,, \tag{A7}$$

so that $f(t+i\beta/4)$ is positive. Another choice suggested by the authors of Ref. [22] is the Metropolis-type filter

$$\hat{f}^a(\nu) = \hat{f}(\nu) = q(\nu)\, e^{-\beta\nu/4} = e^{-\sqrt{1+\beta^2\nu^2}}\, w(\nu/S)\, e^{-\beta\nu/4}\,, \tag{A8}$$

where $q(\nu)$ is supported on $[-S,S]$, and $w(x)$ is a "bump function" with support *only* in the interval $x\in[-1,1]$, for which we use

$$w(x) = \begin{cases} e^{-\frac{1}{5(1-x^2)}} & |x|<1 \\ 0 & |x|\geq 1 \end{cases}.$$

Reference [22, Theorem 34] also proves that this Lindbladian evolution can be simulated on a quantum computer up to time t with time complexity

$$\tilde{\mathcal{O}}(t(\beta+1)|\mathcal{A}|^2 \log^{1+s}(1/\epsilon))\,, \tag{A9}$$

where now ϵ is the precision of the channel in the diamond norm, and $s\geq 1$ is the Gevrey order of the filter function $\hat{f}(\nu)$ (which is for example equal to 1 for the Gaussian filter). This assumes normalisation of the jump operators of the form $\max_{a\in\mathcal{A}}\|A^a\|\leq 1$, access to their block encodings, access to controlled Hamiltonian simulation, and preparation oracles for the filter function $f(t)$ (where $f^a(t)=f(t)$ is taken to be the same for all $a\in\mathcal{A}$) and coherent function $g(t)$.

3. Fermionic Systems and Third Quantisation

We consider a set of fermionic creation and annihilation operators $a_i, a_i^\dagger$, $i\in\{1,\ldots,n\}$. They generate the canonical anti-commutation relations algebra, which is defined by

$$\{a_i,a_j\} = \{a_i^\dagger,a_j^\dagger\} = 0\,,\quad \{a_i,a_j^\dagger\} = \delta_{i,j}\,,$$

where $\{a,b\}=ab+ba$ is the anticommutator. Note that we will often use 1 for the identity operator when the interpretation is obvious from the context. The space of quantum states is the Fock space, which is spanned by $|x\rangle = (a_1^\dagger)^{x_1}\cdots(a_n^\dagger)^{x_n}|0^n\rangle$ where x is a bit string of length n and $|0^n\rangle$ is the vacuum, $a_i|0^n\rangle=0$ for all i. An inner product is defined so that $\langle x|y\rangle=\delta_{x,y}$ and $a^\dagger$ is indeed the adjoint of a. We denote $N_S=\sum_{i\in S}a_i^\dagger a_i$ the number of fermions for a set S of indices. We also denote $N_{\text{tot}}=N_{[n]}$, with $[j]=\{1,\ldots,j\}$. A fermionic operator A is called even if $(-1)^{N_{\text{tot}}}A(-1)^{N_{\text{tot}}}=A$ and odd if $(-1)^{N_{\text{tot}}}A(-1)^{N_{\text{tot}}}=-A$. Physical fermionic Hamiltonians are of the form

$$H = \sum_{I\subseteq[n]} h_I$$

where h_I is an *even* polynomial in $a_i, a_i^\dagger$ with $i\in I$, so that it is even. The Fock space admits similarly an orthogonal decomposition in even and odd orthogonal subspaces — a basis state $|x\rangle$ is even if $|x|$ is even, and odd if $|x|$ is odd, where $|x|$ is the Hamming weight of the bit string. Mathematically, the decomposition into even and odd sectors gives a $\mathbb{Z}_2$ grading. The tensor product $|x\rangle\otimes|y\rangle$ has grading (or parity) $|x|+|y| \mod 2$ and similarly for operators. When we use the tensor product symbol for fermionic objects we implicitly assume this $\mathbb{Z}_2$ grading.

The Fock space has dimension 2^n and can be identified with the space of n qubits. Creation and annihilation operators can be represented on the space of qubits via the Jordan-Wigner transformation

$$N_i = \frac{1}{2}(\mathbf{1}+Z_i)\,,\quad a_i = (-1)^{N_{[i-1]}}\sigma_i^-\,,\quad a_i^\dagger = (-1)^{N_{[i-1]}}\sigma_i^+ \tag{A10}$$

where $\sigma^\pm=(X\pm iY)/2$. Other transformations exist, but we will not need them here. It is also convenient to introduce another basis of the fermionic algebra given by the Majorana operators

$$\omega_{2j-1} = a_j + a_j^\dagger\,,\quad \omega_{2j} = i(a_j - a_j^\dagger)\,,$$

for $j\in[n]$. Note that the ω_i's are self-adjoint and satisfy $\{\omega_i,\omega_j\}=2\delta_{i,j}$.

Next we discuss third quantisation, which is a formalism introduced in [44] that allows one to efficiently solve Lindblad master equations for quadratic fermionic systems. We start by introducing a Hilbert space structure $B\to|B\rangle$ to the space of operators by defining a canonical basis $\{P_\alpha\}_{\alpha\in\{0,1\}^{2n}}$ with $P_\alpha=\prod_{i=1}^{2n}\omega_i^{\alpha_i}$. These basis vectors are orthonormal with respect to the inner product $\langle P|Q\rangle=\frac{1}{4^n}\operatorname{Tr}\!\big(P^\dagger Q\big)$. Now define $2n$ annihilation linear maps c_j over this operator space by $c_j|P_\alpha\rangle=\delta_{\alpha_j,1}|\omega_j P_\alpha\rangle$. The action of their Hermitian

adjoints, called creation linear maps, is readily found to be $c_j^\dagger|P_\alpha\rangle = \delta_{\alpha_j,0}|\omega_j P_\alpha\rangle$. These maps then obey the canonical anticommutation relations, $\{c_j, c_k\} = 0$ and $\{c_j, c_k^\dagger\} = \delta_{j,k}$, and so they act like canonical fermions. They will be referred to as adjoint Fermi maps, or a-fermions for short.

Now observe that

$$\begin{aligned}|P_\alpha\omega_j\rangle &= (-1)^{|\alpha|+\alpha_j}|\omega_j P_\alpha\rangle \\ |\omega_j\omega_k P_\alpha\rangle - |P_\alpha\omega_j\omega_k\rangle &= 2(c_j c_k^\dagger + c_j^\dagger c_k)|P_\alpha\rangle \\ |\omega_j P_\alpha\rangle &= (c_j^\dagger + c_j)|P_\alpha\rangle \\ (-1)^{\alpha_j}|\omega_j P_\alpha\rangle &= (c_j^\dagger - c_j)|P_\alpha\rangle \\ (-1)^{|\alpha|}|P_\alpha\rangle &= \exp(i\pi N)|P_\alpha\rangle,\end{aligned}$$

where $N = \sum_j c_j^\dagger c_j$ is the number operator. Also note that the Lindbladian, while not necessarily conserving the number of Majorana fermions, conserves their parity; and so we can restrict ourselves to the physical case of even numbers of Majorana fermions, hence recognizing that $\exp(i\pi N) = 1$ on this subspace. These properties hence allow us to rewrite the action of a quadratic fermionic Lindbladian like

$$\mathcal{L}^\dagger|_+[P_\alpha] \cong \mathcal{L}^\dagger|_+|P_\alpha\rangle$$

by expressing $\mathcal{L}^\dagger|_+$ as a quadratic form in a-fermions. Hence the spectrum of $\mathcal{L}^\dagger|_+$ can then be simply studied as that of a quadratic (not necessarily Hermitian) fermionic system. We shall do this explicitly in Section B 1.

Example A.6. *Consider a simple fermionic superoperator $\mathcal{L}[\rho] = a\cdot\omega_1\rho\omega_2 + b\cdot\omega_1\omega_2\rho + c\cdot\rho\omega_1\omega_2$. Associating the Hilbert space structure to this space, we can write $\mathcal{L}|\rho\rangle = a|\omega_1\rho\omega_2\rangle + b|\omega_1\omega_2\rho\rangle + c|\rho\omega_1\omega_2\rangle$. Applying the second rule to the last term, we get $\mathcal{L}|\rho\rangle = a|\omega_1\rho\omega_2\rangle + b|\omega_1\omega_2\rho\rangle + c(|\omega_1\omega_2\rho\rangle - 2(c_1c_2^\dagger + c_1^\dagger c_2)|\rho\rangle)$. Now applying the first rule to the first term, we obtain $\mathcal{L}|\rho\rangle = a(-1)^{|\alpha|+\alpha_2}|\omega_1\omega_2\rho\rangle + (b+c)|\omega_1\omega_2\rho\rangle - 2c(c_1c_2^\dagger + c_1^\dagger c_2)|\rho\rangle$. Restricting our view to physical states with $|\alpha|$ being even, and applying rules 3 and 4, we finally arrive at $\mathcal{L}|_+|\rho\rangle = a(c_1^\dagger + c_1)(c_2^\dagger - c_2)|\rho\rangle + (b+c)(c_1^\dagger + c_1)(c_2^\dagger + c_2)|\rho\rangle - 2c(c_1c_2^\dagger + c_1^\dagger c_2)|\rho\rangle$. Hence we see that*

$$\begin{aligned}\mathcal{L}|_+ &\cong a(c_1^\dagger + c_1)(c_2^\dagger - c_2) + (b+c)(c_1^\dagger + c_1)(c_2^\dagger + c_2) - 2c(c_1c_2^\dagger + c_1^\dagger c_2) \\ &= a(c_1^\dagger + c_1)(c_2^\dagger - c_2) + b(c_1^\dagger + c_1)(c_2^\dagger + c_2) + c(c_1^\dagger - c_1)(c_2^\dagger - c_2)\,.\end{aligned}$$

Remark A.7. The idea of Section B 2, where we will prove stability of the gap of this Lindbladian under perturbation, is to view both the unperturbed part and the perturbation of the corresponding parent Hamiltonian in third quantisation. Since the unperturbed part will transform into a free fermionic Hamiltonian, we will be able to use gap stability results for free fermions [46–48] to show constant gap of the interacting Lindbladian.

4. On Locality

Reference [22] shows that if H is a geometrically local Hamiltonian, A_a are local and the filter function is Gaussian, then the Lindblad operators L_a are quasi-local and G is a sum of quasi-local terms. Here we extend this result and discuss the locality properties of the parent Hamiltonian for fermionic systems and systems with exponentially decaying interactions. The quasi-locality of the parent Hamiltonian will be an important ingredient in the proofs of gap stability we present below.

We consider a lattice Λ. For qubit systems, an operator O has support I if it can be written as $O = \mathbf{1}_{\Lambda\setminus I}\otimes A$ for some operator A acting on the space of the qubits at I. This implies that operators that are spatially separated — i.e. with disjoint supports — commute. This definition is not useful for fermionic systems as odd fermionic operators anti-commute even if they are spatially separated. We say that a fermionic operator A has support I if A is a polynomial in $a_i, a_i^\dagger$ with $i \in I$. We call a fermionic operator local if its support is a geometrically local region of the lattice, and we call a fermionic operator quasi-local if it can be approximated by a local operator with an exponentially decaying error. The following result is a generalisation of [22, Prop. 20] for Hamiltonians with exponentially decaying interactions.

Proposition A.8. *Consider a Hamiltonian H with interactions that decay at least exponentially, and local jump operators A^a with Gaussian filter functions. Then the parent Hamiltonian (4.1) is a sum of quasi-local terms.*

Proof. We define local approximations of $\tilde{L}, \tilde{G}$, and L from (A5) and (A6) by

$$\tilde{L}_a^{(r)} = \int_{-\infty}^{\infty} f^a(t+i\beta/4) e^{iH_{B_r(a)}t} A^a e^{-iH_{B_r(a)}t}\, \mathrm{d}t\,,$$

$$\tilde{G}^{(r)} = \sum_{a\in\mathcal{A}} \tilde{G}_a^{(r)} = \sum_{a\in\mathcal{A}} \int_{-\infty}^{\infty} g(t+i\beta/4) e^{iH_{B_r(a)}t} (L_a^{(r)\dagger} L_a^{(r)}) e^{-iH_{B_r(a)}t}\, \mathrm{d}t\,,$$

$$L_a^{(r)} = \int_{-\infty}^{\infty} f^a(t) e^{iH_{B_r(a)}t} A^a e^{-iH_{B_r(a)}t}\, \mathrm{d}t\,,$$

where $B_r(a)$ is a ball of radius r around the support of A^a and $H_\Omega = \sum_{I\,|\,I\cap\Omega\neq\emptyset} h_I$ is the truncated Hamiltonian to region Ω.

Here we shall use a weaker version of the Lieb-Robinson bound than the one for local systems [60, Lemma 5] used in [22, Prop. 20], which also holds for exponentially decaying Hamiltonian interactions, and tells us that

$$\left\| e^{iHt} A^a e^{-iHt} - e^{iH_{B_r(a)}t} A^a e^{-iH_{B_r(a)}t} \right\| \le \|A^a\| \min\left\{2, Je^{-\mu r}(e^{\mu v|t|}-1)\right\}$$

for some constants J, v, and μ. From here, we shall assume $\|A^a\| \le 1$. Using the Gaussian filter (A7), it follows that

$$\|L_a - L_a^{(r)}\| \le \int_{-\infty}^{\infty} |f(t)| Je^{-\mu r}(e^{\mu v|t|}-1)\, \mathrm{d}t = Ce^{-\mu r}\,,$$

and similarly that

$$\|\tilde{L}_a - \tilde{L}_a^{(r)}\| \le \int_{-\infty}^{\infty} |f(t+i\beta/4)| Je^{-\mu r}(e^{\mu v|t|}-1)\, \mathrm{d}t = \tilde{C}e^{-\mu r}\,,$$

as the integrals over $|f(t)|e^{c|t|}$ and $|f(t+i\beta/4)|e^{c|t|}$ converge.

Regarding the coherent term, consider the function $\hat{g}(\nu) = \frac{i}{2}\tanh\left(\frac{\beta\nu}{4}\right)$ without the presence of the bump function. Its representation in the time domain is then $g(t) = \frac{1}{\beta}\frac{1}{\sinh(2\pi t/\beta)}$, which decays exponentially as $|t| \to \infty$ but has a singularity at $t = 0$. But the coherent term in the parent Hamiltonian then depends on $g(t+i\beta/4) = -\frac{i}{\beta}\frac{1}{\cosh(2\pi t/\beta)}$, which is no longer singular at $t = 0$. Hence we can observe that

$$\begin{aligned}
\|\tilde{G}_a - \tilde{G}_a^{(r)}\| &\le \int_{-\infty}^{\infty} |g(t+i\beta/4)| \left\| e^{iHt} L_a^\dagger L_a e^{-iHt} - e^{iH_{B_r(a)}t} L_a^{(r)\dagger} L_a^{(r)} e^{-iH_{B_r(a)}t} \right\| \mathrm{d}t \\
&\le 2\int_{-\infty}^{\infty} |g(t+i\beta/4)| \left\| e^{iHt} L_a e^{-iHt} - e^{iH_{B_r(a)}t} L_a^{(r)} e^{-iH_{B_r(a)}t} \right\| \mathrm{d}t \\
&\le 2\int_{-\infty}^{\infty}\int_{-\infty}^{\infty} |g(t+i\beta/4)| \cdot |f(s)| \left\| e^{iH(t+s)} A^a e^{-iH(t+s)} - e^{iH_{B_r(a)}(t+s)} A^a e^{-iH_{B_r(a)}(t+s)} \right\| \mathrm{d}s\mathrm{d}t \\
&\le 2\int_{-\infty}^{\infty}\int_{-\infty}^{\infty} |g(t+i\beta/4)| \cdot |f(s)| \min\left\{2, Je^{-\mu r}(e^{\mu v|t+s|}-1)\right\} \mathrm{d}s\mathrm{d}t \\
&\le 2\|f\|_1 \int_{-\infty}^{\infty} |g(t+i\beta/4)| \min\left\{2, Je^{-\mu r}(ce^{\mu v|t|}-1)\right\} \mathrm{d}t\,,
\end{aligned}$$

where the last inequality follows from splitting $|t+s| \le |t| + |s|$ and carrying out the integral over s, which converges since $f(s)$ is Gaussian. Now this minimum changes at $|t| = t^* = \frac{1}{\mu v}\log\left((2e^{\mu r}/J+1)/c\right)$, which we can lower bound by $t^* \ge \frac{1}{\mu v}\log(2/(cJ)) + r/v = \tilde{c} + r/v$. Hence we can continue the upper bound like

$$\begin{aligned}
\frac{\|\tilde{G}_a - \tilde{G}_a^{(r)}\|}{\|f\|_1} &\le 2\int_{|t|>\tilde{c}+r/v} |g(t+i\beta/4)| \cdot 2\, \mathrm{d}t + 2\int_{|t|<\tilde{c}+r/v} |g(t+i\beta/4)| \cdot Je^{-\mu r}(ce^{\mu v|t|}-1)\, \mathrm{d}t \\
&= \frac{8}{\beta}\int_{\tilde{c}+r/v}^{\infty} \frac{1}{\cosh(2\pi t/\beta)}\, \mathrm{d}t + \frac{4J}{\beta}e^{-\mu r}\int_0^{\tilde{c}+r/v} \frac{1}{\cosh(2\pi t/\beta)}(ce^{\mu vt}-1)\, \mathrm{d}t \\
&\le \frac{16}{\beta}\int_{\tilde{c}+r/v}^{\infty} e^{-2\pi t/\beta}\, \mathrm{d}t + \frac{8J}{\beta}e^{-\mu r}\int_0^{\tilde{c}+r/v} e^{-2\pi t/\beta}(ce^{\mu vt}-1)\, \mathrm{d}t \\
&= \frac{8}{\pi}e^{-2\pi(\tilde{c}+r/v)/\beta} + \frac{4J}{\pi}e^{-\mu r}\left(e^{-2\pi(\tilde{c}+r/v)/\beta}-1\right) - \frac{8cJ}{2\pi-\beta\mu v}\left(e^{-2\pi r/(v\beta)+\tilde{c}(\mu v-2\pi/\beta)} - e^{-\mu r}\right)\,,
\end{aligned}$$

which is indeed exponentially decaying in r, proving that $\tilde{G}_a$ is quasi-local, and hence that $\tilde{G}$ is a sum of quasi-local terms. Altogether this shows that the parent Hamiltonian $\mathcal{H}$ is a sum of quasi-local terms. □

One can observe that, since the transformation to the parent Hamiltonian improves the decay of the filter functions and gets rid of the singularity of the coherent function $g(t)$ appearing in the defining integrals, the parent Hamiltonian is actually a better behaved and a more natural object than the Lindbladian itself; even though it contains terms of the form $\sigma_\beta^{-1/4} O \sigma_\beta^{1/4}$, which are generally non-local and can grow to infinite size even for a finite β [66], as we do not have Lieb-Robinson bounds for imaginary/Euclidean time evolution.

Note that the Lieb-Robinson bound, which follows from the bound on the commutator with the Hamiltonian terms, is true in our fermionic setting independently of whether A_a is even or odd in the number of fermions, since the constituent Hamiltonian terms are always even, so that (4.2) still holds. We refer to [61] for more on Lieb-Robinson bounds and locality for fermions.

We conclude this section with a remark on the runtime of the quantum algorithm that simulates the Lindbladian dynamics. Note that if we take local jump operators we have $|\mathcal{A}| = \Omega(n)$. This is due to quasi-locality of L_a's and G, and the irreducibility criterion for uniqueness of the stationary state σ_β discussed in section A 1 that requires the L_a's to span the whole operator algebra. This implies that the runtime of Eq. (A9) is lower bounded by $\Omega(n^2)$ even before considering the mixing time.

5. Fermi-Hubbard Model

As mentioned in the overview (Section I), this work is concerned with the applicability of a particular quantum Gibbs sampler to fermionic systems, and specifically to the Fermi-Hubbard model. In its original form, it consists of fermions on a D-dimensional lattice and is governed by the Hamiltonian

$$H_{\rm FH} = -t \sum_{\langle i,j\rangle,\sigma} \left(a_{i,\sigma}^\dagger a_{j,\sigma} + a_{j,\sigma}^\dagger a_{i,\sigma} \right) + U \sum_i a_{i,\uparrow}^\dagger a_{i,\uparrow} a_{i,\downarrow}^\dagger a_{i,\downarrow}, \tag{A11}$$

where $\langle \cdot, \cdot \rangle$ means neighbouring sites on the lattice, $\sigma \in \{\uparrow, \downarrow\}$, and $a_{i,\sigma}^{(\dagger)}$ are the usual fermionic annihilation (creation) operators on site i with spin σ. The model parameters t and U are usually positive, though we will also consider $U < 0$ in some instances (the attractive Fermi-Hubbard model).

There is also a *spinless* (sometimes called *polarised*) version of this model, which removes the spin from the particles and replaces the on-site interaction with that of nearest neighbours. Its Hamiltonian is therefore

$$H_{\rm pFH} = -t \sum_{\langle i,j\rangle} \left(a_i^\dagger a_j + a_j^\dagger a_i \right) + U \sum_{\langle i,j\rangle} a_i^\dagger a_i a_j^\dagger a_j. \tag{A12}$$

Being less computationally demanding on classical hardware (for the same number of sites) but still exhibiting interesting behaviour, the spinless Fermi-Hubbard model is a good candidate for numerical finite-size study of the quantum Gibbs samplers considered in this work, and in Section C we present results for both the spinful and spinless Fermi-Hubbard models.

Appendix B: Analytical Results on Interacting fermions

This chapter will provide the bulk of the proof for gapness of the Lindbladian $\mathcal{L}^\dagger$ corresponding to weakly interacting fermionic systems, and hence for the efficiency of the quantum Gibbs state preparation. In Section B 1, we will explicitly calculate the gap of the Lindbladian for free fermions and express it using the third quantisation as a quadratic fermionic system. Section B 2 will then bound the perturbation of the Lindbladian for the interacting fermionic case, and explain how we can use the stability of free fermions to lower bound the gap. In Section B 3, we discuss the atomic limit where inter site interactions are set to zero and show that the Lindbladian gap persists also for perturbations around this limit. Finally, Section B 4 discusses how these results on the gap translate to mixing time and algorithmic complexity of Gibbs state preparation.

1. Spectrum of the Lindbladian for Free Fermions

Lemma B.1. *For a free fermionic system, given by $H_0 = \boldsymbol{\omega}^T \cdot h \cdot \boldsymbol{\omega} = \sum_{i,j} \omega_i h_{ij} \omega_j$ with h Hermitian and anti-symmetric, by taking the set of jump operators to be $\mathbf{A} = M \cdot \boldsymbol{\omega}$, where M is a unitary matrix, and the filter functions $\hat{f}^a$ to be real and equal, the coherent term G vanishes.*

Proof. Note that on the space $\mathcal{S} = \operatorname{span}\{\omega_a\}$, we have that $\operatorname{ad}_{H_0} =_{\mathcal{S}} -4h$. Hence the time-evolved jump operators are given by

$$\mathbf{A}(t) = e^{iH_0 t} \mathbf{A} e^{-iH_0 t} = e^{iH_0 t} M \cdot \boldsymbol{\omega} e^{-iH_0 t} = M \cdot e^{-4iht} \cdot \boldsymbol{\omega}\,,$$

and the Lindblad operators are then just

$$\mathbf{L} = M \cdot \int_{-\infty}^{\infty} f(t) e^{-4iht} \, \mathrm{d}t \cdot \boldsymbol{\omega} = M \cdot \hat{f}(-4h) \cdot \boldsymbol{\omega}\,.$$

Hence we get that

$$\begin{aligned}\sum_{a\in\mathcal{A}} L_a^\dagger L_a = \mathbf{L}^\dagger \cdot \mathbf{L} &= \boldsymbol{\omega}^T \cdot \hat{f}(-4h) \cdot M^\dagger \cdot M \cdot \hat{f}(-4h) \cdot \boldsymbol{\omega} \\ &= \boldsymbol{\omega}^T \cdot [\hat{f}(-4h)]^2 \cdot \boldsymbol{\omega} \\ &= \boldsymbol{\omega}^T \cdot \left(\frac{[\hat{f}(-4h)]^2 - [\hat{f}(4h)]^2}{2} + \mathrm{diag}\left([\hat{f}(-4h)]^2\right) \right) \cdot \boldsymbol{\omega} \\ &= \boldsymbol{\omega}^T \cdot q(4h)^2 \cdot \sinh(2\beta h) \cdot \boldsymbol{\omega} + \mathrm{Tr}\left(\hat{f}(-4h)^2\right)\,,\end{aligned}$$

where we have split up the matrix $[\hat{f}(-4h)]^2$ into its anti-symmetric, diagonal, and a symmetric hollow part. Now observe that

$$[(H_0)_n, \boldsymbol{\omega}^T \cdot A \cdot \boldsymbol{\omega}] = \boldsymbol{\omega}^T \cdot 4^n [(h)_n, A] \cdot \boldsymbol{\omega}$$

for any anti-symmetric matrix A, where $[(X)_n, Y]$ denotes the n-th iterated commutator. Hence by using the Campbell identity, we obtain

$$\begin{aligned}\sum_{a\in\mathcal{A}} e^{iH_0t} L_a^\dagger L_a e^{-iH_0t} &= \boldsymbol{\omega}^T \cdot e^{4ith} \cdot q(4h)^2 \cdot \sinh(2\beta h) \cdot e^{-4ith} \cdot \boldsymbol{\omega} + \mathrm{Tr}\left(\hat{f}(-4h)^2\right) \\ &= \boldsymbol{\omega}^T \cdot q(4h)^2 \cdot \sinh(2\beta h) \cdot \boldsymbol{\omega} + \mathrm{Tr}\left(\hat{f}(-4h)^2\right)\,,\end{aligned}$$

independent of t, proving that $\sum_{a\in\mathcal{A}} L_a^\dagger L_a$ is an integral of motion under H_0, which means that

$$G = \int_{-\infty}^{\infty} g(t) \cdot \sum_{a\in\mathcal{A}} e^{iH_0t} L_a^\dagger L_a e^{-iH_0t} \, \mathrm{d}t = \int_{-\infty}^{\infty} g(t) \cdot \sum_{a\in\mathcal{A}} L_a^\dagger L_a \, \mathrm{d}t \propto \hat{g}(0) = 0\,,$$

meaning that the coherent term vanishes. □

Proposition B.2. *The Lindbladian $\mathcal{L}_0^\dagger$ corresponding to the free fermionic Hamiltonian H_0 with the set of jump operators $\{\omega_a\}_{a=1}^{2n}$ and equal real filter functions $\hat{f}^a(\nu) = \hat{f}(\nu) = q(\nu)e^{-\beta\nu/4}$ has spectral gap given by*

$$\Delta_0 = 2 \cdot \min_i q(4\epsilon_i)^2 \cosh(2\beta\epsilon_i),$$

where $\epsilon_i \in \mathrm{spec}(h)$ are the eigenvalues of the single particle Hamiltonian h.

Proof. Note that by spectral gap we mean the gap between the highest and second highest eigenvalue of the Lindbladian, i.e. the one that bounds the mixing time; though this will turn out to be the same gap as between the lowest and second lowest eigenvalue.

For future convenience, let's consider the similarity transformation $\mathcal{H}_0[\rho] = \sigma_\beta^{-1/4} \cdot \mathcal{L}_0^\dagger[\sigma_\beta^{1/4} \cdot \rho \cdot \sigma_\beta^{1/4}] \cdot \sigma_\beta^{-1/4}$ into the parent Hamiltonian, which is Hermitian due to the QDB condition, i.e. self-adjoint w.r.t. the Hilbert-Schmidt inner product. As this is a similarity transformation, the spectrum of this superoperator will be the same as of $\mathcal{L}_0^\dagger$. We can calculate that

$$\sigma_\beta^{-1/4} L_a \sigma_\beta^{1/4} = \hat{f}(-4h)_a \cdot e^{-\beta h} \cdot \boldsymbol{\omega},$$

and the QDB condition also ensures $\sigma_\beta^{-1/4} L_a \sigma_\beta^{1/4} = \sigma_\beta^{1/4} L_a^\dagger \sigma_\beta^{-1/4}$. Using the calculation from Lemma B.1, we can also straightforwardly evaluate

$$\begin{aligned}\sum_{a\in\mathcal{A}} \sigma_\beta^{-1/4} L_a^\dagger L_a \sigma_\beta^{1/4} &= \boldsymbol{\omega}^T \cdot q(4h)^2 \cdot \sinh(2\beta h) \cdot \boldsymbol{\omega} + \mathrm{Tr}\left(\hat{f}(-4h)^2\right) \\ &= \sum_{a\in\mathcal{A}} \sigma_\beta^{1/4} L_a^\dagger L_a \sigma_\beta^{-1/4}\,,\end{aligned}$$

and so the parent Hamiltonian simplifies to

$$\begin{aligned}\mathcal{H}_0[\rho] = \sum_{a\in\mathcal{A}} \boldsymbol{\omega}^T \cdot q(4h)_a^\dagger \cdot \rho \cdot q(4h)_a \cdot \boldsymbol{\omega} - \frac{1}{2}\boldsymbol{\omega}^T \cdot q(4h)^2 \cdot \sinh(2\beta h) \cdot \boldsymbol{\omega} \cdot \rho - \rho \cdot \boldsymbol{\omega}^T \cdot \frac{1}{2} q(4h)^2 \cdot \sinh(2\beta h) \cdot \boldsymbol{\omega} \\ - \mathrm{Tr}\left(q(4h)^2 \cdot \cosh(2\beta h)\right) \cdot \rho\,.\end{aligned}$$

Now following Prosen's third quantisation [44], which we reviewed in Section A 3, we obtain the equivalent form

$$\mathcal{H}_0 \cong -\mathbf{c}^\dagger \cdot S \cdot \mathbf{c} + \mathbf{c} \cdot S \cdot \mathbf{c}^\dagger + \mathbf{c}^\dagger \cdot A \cdot \mathbf{c}^\dagger + \mathbf{c} \cdot A \cdot \mathbf{c} - \mathrm{Tr}\left(\sqrt{S^2 + A^2}\right) ,$$

where we have restricted the Hilbert space to that of physical states with even numbers of Majorana fermions; and $S = q(4h)^2$, $A = q(4h)^2 \sinh(2\beta h)$, and $\{c_i^\dagger, c_i\}_{i=1}^{2n}$ is a set of $2n$ canonical fermionic creation and annihilation operators. This is just a quadratic a-fermionic system with dynamical matrix $D = \begin{pmatrix} -S & A \\ A & S \end{pmatrix}$. Since both S and A are just functions of h, they are simultaneously diagonalisable with the eigenbasis of h, and hence D is also easily diagonalisable, with eigenvalues

$$\lambda_i^\pm = \pm q(4\epsilon_i)^2 \cosh(2\beta\epsilon_i) ,$$

where $\epsilon_i \in \mathrm{spec}(h)$. Finally, the complete spectrum of $\mathcal{H}_0$, which is the same as that of $\mathcal{L}_0^\dagger$, is then

$$\mathrm{spec}(\mathcal{L}_0^\dagger) = \left\{ \sum_{i=1}^{2n} (-1 + (-1)^{x_i}) \cdot q(4\epsilon_i)^2 \cosh(2\beta\epsilon_i) \right\}_{x \in \{0,1\}^{2n}} ,$$

and the corresponding spectral gap is

$$\Delta_0 = 2 \cdot \min_i q(4\epsilon_i)^2 \cosh(2\beta\epsilon_i) .$$

This argument also assures that the Gibbs state is the unique fixed point of the dynamics generated by $\mathcal{L}_0^\dagger$. □

Proposition B.3. *For free fermionic Hamiltonians, which have a bounded single particle Hamiltonian — meaning $\|h\| \leq \mathcal{O}(1)$ — when taking the initial state to be a Gaussian state ρ_0, the Lindbladian $\mathcal{L}_0^\dagger$ mixes rapidly, i.e. in logarithmic time, with an upper bound*

$$t_{\mathrm{mix}} \leq \frac{1}{2\Delta_0} \log\left(\frac{2n}{\epsilon}\right) = \frac{1}{4 \min_i q(4\epsilon_i)^2 \cosh(2\beta\epsilon_i)} \log\left(\frac{2n}{\epsilon}\right) .$$

Proof. First, we need to recognise that when we start with a Gaussian state ρ_0 and evolve it with a quadratic Lindbladian, we will stay within the subspace of Gaussian states. These can be uniquely characterised by their covariance matrices $\Gamma_{ij} = \frac{i}{2}\mathrm{Tr}([\omega_i, \omega_j]\rho)$. Denoting $\Gamma(t)$ the covariance matrix of $\rho(t) = e^{t\mathcal{L}^\dagger}[\rho_0]$, we can follow [59] to obtain its equation of motion generated by our Lindbladian as

$$\frac{\mathrm{d}}{\mathrm{d}t}\Gamma(t) = -2q(4h)^2\cosh(2\beta h) \cdot \Gamma(t) - \Gamma(t) \cdot 2q(4h)^2 \cosh(2\beta h) + 2iq(4h)^2 \sinh(2\beta h) .$$

Assume that the initial covariance matrix is Γ_0, then we can straightforwardly solve this ODE with

$$\Gamma(t) = e^{-2q(4h)^2\cosh(2\beta h)\cdot t} \cdot \left(\Gamma_0 - \frac{i}{2}\tanh(2\beta h)\right) \cdot e^{-2q(4h)^2\cosh(2\beta h)\cdot t} + \frac{i}{2}\tanh(2\beta h) .$$

We can also check that the covariance matrix of the Gibbs state σ_β is $\frac{i}{2}\tanh(2\beta h) = \Gamma(\infty)$, and so the evolution indeed converges to the Gibbs state.

Finally, we can use optimal trace norm bounds obtained in [45], which tell us that

$$\begin{aligned}
\left\| e^{t\mathcal{L}^\dagger}[\rho_0] - \sigma_\beta \right\|_{\mathrm{Tr}} &\leq \frac{1}{2}\|\Gamma(t) - \Gamma_{\sigma_\beta}\|_{\mathrm{Tr}} \\
&= \frac{1}{2}\left\| e^{-2q(4h)^2\cosh(2\beta h)\cdot t} \cdot \left(\Gamma_0 - \frac{i}{2}\tanh(2\beta h)\right) \cdot e^{-2q(4h)^2\cosh(2\beta h)\cdot t} \right\|_{\mathrm{Tr}} \\
&\leq \frac{1}{2}\left\| e^{-2q(4h)^2\cosh(2\beta h)\cdot t}\right\|^2 \cdot \left\|\Gamma_0 - \frac{i}{2}\tanh(2\beta h)\right\|_{\mathrm{Tr}} \\
&= \frac{1}{2}e^{-2\Delta_0\cdot t} \cdot \left\|\Gamma_0 - \frac{i}{2}\tanh(2\beta h)\right\|_{\mathrm{Tr}} \\
&\leq 2n \cdot e^{-2\Delta_0 \cdot t} \\
&\overset{\mathrm{set}}{\leq} \epsilon\ .
\end{aligned}$$

This final inequality can be then solved for t like $t \geq \frac{1}{4\min_i q(4\epsilon_i)^2\cosh(2\beta\epsilon_i)}\log\left(\frac{2n}{\epsilon}\right)$, and hence we deduce that

$$t_{\mathrm{mix}} \leq \frac{1}{4 \min_i q(4\epsilon_i)^2 \cosh(2\beta\epsilon_i)} \log\left(\frac{2n}{\epsilon}\right) .$$

□

Corollary B.3.1. *Taking the initial state to be a convex combination of Gaussian states, $\rho_0 = \sum_i \alpha_i \rho_i^{(\mathrm{Gauss})}$, where α_i are positive and sum up to 1, we also get rapid mixing in time*

$$t_{\mathrm{mix}} \leq \frac{1}{2\Delta_0} \log\left(\frac{2n}{\epsilon}\right).$$

Proof. We have that

$$\begin{aligned} \|e^{t\mathcal{L}^\dagger}[\rho_0] - \sigma_\beta\|_{\mathrm{Tr}} &= \left\|\sum_i \alpha_i e^{t\mathcal{L}^\dagger}\left[\rho_i^{(\mathrm{Gauss})}\right] - \sigma_\beta\right\|_{\mathrm{Tr}} = \left\|\sum_i \alpha_i \left(e^{t\mathcal{L}^\dagger}\left[\rho_i^{(\mathrm{Gauss})}\right] - \sigma_\beta\right)\right\|_{\mathrm{Tr}} \\ &\leq \sum_i \alpha_i \left\|e^{t\mathcal{L}^\dagger}\left[\rho_i^{(\mathrm{Gauss})}\right] - \sigma_\beta\right\|_{\mathrm{Tr}} \\ &\leq \sum_i \alpha_i \cdot 2n \cdot e^{-2\Delta_0 \cdot t} = 2n \cdot e^{-2\Delta_0 \cdot t}. \end{aligned}$$

□

Corollary B.3.2. *For free fermionic Hamiltonians, which have a bounded single particle Hamiltonian — meaning $\|h\| \leq \mathcal{O}(1)$ — the Lindbladian $\mathcal{L}_0^\dagger$ has a constant spectral gap Δ_0 and is efficiently simulable.*

Proof. Using for example the Gaussian filter function $\hat{f}(\nu) = e^{-(\beta\nu+1)^2/8+1/8}$, which is efficiently implementable, the gap simplifies to

$$\Delta_0 = 2 \cdot e^{-4\beta^2\|h\|^2} \cosh(2\beta\|h\|), \tag{B1}$$

a monotonically decreasing function w.r.t. $\|h\|$, which is hence bounded below when $\|h\| \leq \mathcal{O}(1)$. Such a condition is assured when considering free fermionic Hamiltonians with hopping rates decaying at least polynomially, as then $\|h\|_\infty \leq \mathcal{O}(1)$. Here the induced infinity norm means the maximal absolute row sum of the matrix. The mixing time is then $t_{\mathrm{mix}} = \mathcal{O}(\log(n/\epsilon))$ and the total time complexity of the algorithm will be $\tilde{\mathcal{O}}(n^2 e^{4\beta^2\|h\|^2} \operatorname{polylog}(1/\epsilon))$, where ϵ is the required precision from the Gibbs state in the trace norm. The details about complexity will be discussed later in Section B 4. □

2. Stability of the Free Fermionic Gap Under Perturbations

In this section, we shall consider the Lindbladian $\mathcal{L}_0^\dagger$ corresponding to a quasi-local free fermionic Hamiltonian $H_0 = \sum_{i,j} \omega_i h_{ij} \omega_j$, and the Lindbladian $\mathcal{L}^\dagger$ corresponding to the perturbed quasi-local fermionic Hamiltonian $H = H_0 + \lambda V$. We will denote their (Hermitian) parent Hamiltonians, obtained via similarity transformations, by $\mathcal{H}_0$ and $\mathcal{H}$ respectively; and the perturbation of the parent Hamiltonians by $\mathcal{V} = \mathcal{H} - \mathcal{H}_0$. We shall prove that $\mathcal{L}^\dagger$ remains gapped for perturbations with strength $|\lambda| \leq \lambda_{\max}$ for some constant $\lambda_{\max}$ by using theorems about stability of the gap for lattice fermions proved in [46] and refined in [47, 48].

Definition B.4 (Definition 1 of [46]). *An operator W is said to have (K, μ)-decay if it can be decomposed as*

$$W = \sum_{r \geq 1} \sum_{C \in \mathcal{C}(r)} W_C,$$

where $\mathcal{C}(r)$ denotes the set of cubes with side length r, and W_C are operators supported only on cubes C such that

$$\max_{C \in \mathcal{C}(r)} \|W_C\| \leq K e^{-\mu r}$$

with positive constants K and μ.

Definition B.5 (Definition 2 of [46]). *An operator $B = \sum_{i,j\in\Lambda} \omega_i B_{ij} \omega_j$ is said to have a $[J, \nu]$-decay if*

$$|B_{ij}| \leq J e^{-\nu \operatorname{dist}(i,j)}$$

with positive constants J and ν, where $\operatorname{dist}(i,j)$ is the distance on Λ in Manhattan metric.

Theorem B.6 (Corollary 1 of [46]). *If the Hermitian operator $\mathcal{H}_{\mathrm{free}} = \sum_{i,j\in\Lambda} \omega_i \mathfrak{H}_{ij} \omega_j$ has $[J, \nu]$-decay and a spectral gap Δ_0, and the Hermitian operator $\mathcal{H}_{\mathrm{int}}$ has (K, μ)-decay, then there exist positive constants $K_{\max}$ and s independent of the system size, such that whenever $K \leq K_{\max}$, the gap of $\mathcal{H}_{\mathrm{free}} + \mathcal{H}_{\mathrm{int}}$ is lower bounded by $\Delta_0 - sK$.*

Lemma B.7. *Assume that H_0 has $[J_0,\nu_0]$-decay, and that we are using the Gaussian filter function with Majorana jump operators. Then the parent Hamiltonian $\mathcal{H}_0$ of the Lindbladian $\mathcal{L}_0^\dagger$ corresponding to the free fermionic system simplifies to a free fermionic Hamiltonian with $[J,\nu]-$decay using the third quantisation.*

Proof. Here we are considering the transformed operator $\mathcal{H}_0[\rho] = \sigma_{\beta,0}^{-1/4}\cdot\mathcal{L}_0^\dagger[\sigma_{\beta,0}^{1/4}\cdot\rho\cdot\sigma_{\beta,0}^{1/4}]\cdot\sigma_{\beta,0}^{-1/4}$, which is Hermitian due to the QDB condition. The simplification to free fermions was shown in Proposition B.2, from which we may further define the new Majorana modes to match the Definition B.5. The quasi-locality of the parent Hamiltonians for systems with exponentially decaying interactions was shown in Proposition 4.

Alternatively, we can study the locality of $\mathcal{H}_0$ directly by considering the decay of elements of the matrices $S = q(4h)^2$ and $A = q(4h)^2\sinh(2\beta h)$, which give its a-fermionic description, where $q(\nu) = e^{-\beta^2\nu^2/8}$, i.e. $q(4h)^2 = e^{-4\beta^2h^2}$. Note that h^2 is a positive semi-definite, real, Hermitian matrix; which has bounded eigenvalues, as $\|h\|_\infty \le \mathcal{O}(1)$. Hence we can use [67, Theorem 3.1] to say that

$$|S_{ij}| = \left|[q(4h)^2]_{ij}\right| \le \exp(-\mathcal{O}(\mathrm{dist}(i,j)))\,,$$

where $\mathrm{dist}(i,j)$ represents the distance on the adjacency graph of the matrix h^2. For short-range Hamiltonians, this then shows explicitly that S is quasi-local. A follows similarly as $A = \frac{e^{1/4}}{2}(e^{-4(\beta h-1/4)^2} - e^{-4(\beta h+1/4)^2})$, and we can apply the same theorem to these two parts separately. This shows directly that $\mathcal{H}_0$ is in the a-fermionic picture quasi-local for $(k,l)-$local Hamiltonians H_0. □

Lemma B.8. *Assume further that the interaction term V consists only of terms with even number of Majorana fermions, and that the interactions decay at least exponentially as described in Section A 4. Then the perturbation of the parent Hamiltonian, $\mathcal{V} = \mathcal{H}-\mathcal{H}_0$, has (K,μ)-decay for some constants K and μ, where K is upper bounded by $c|\lambda|$, with c being a constant independent of the system size.*

Proof. Note that $\mathcal{V}$ is Hermitian, as both $\mathcal{H}$ and $\mathcal{H}_0$ are due to their respective detailed-balance conditions. Its explicit form together with detailed calculations for this proof are in Section E.

To prove the $(c|\lambda|,\mu)$-decay of $\mathcal{V}$, we shall start by considering $\|\sigma_\beta^{-1/4}L_a\sigma_\beta^{1/4} - \sigma_{\beta,0}^{-1/4}L_a^0\sigma_{\beta,0}^{1/4}\|$ and show it is upper bounded by $c_1|\lambda|$:

$$\|\sigma_\beta^{-1/4}L_a\sigma_\beta^{1/4} - \sigma_{\beta,0}^{-1/4}L_a^0\sigma_{\beta,0}^{1/4}\| \le \int_{-\infty}^{\infty}|f^a(t)|\left\|e^{H(\beta/4+it)}A^ae^{-H(\beta/4+it)} - e^{H_0(\beta/4+it)}A^ae^{H_0(\beta/4+it)}\right\|\,\mathrm{d}t$$

Now we can use Lemma D.1 to say that

$$\begin{aligned}\left\|e^{H(\beta/4+it)}A^ae^{-H(\beta/4+it)} - e^{H_0(\beta/4+it)}A^ae^{H_0(\beta/4+it)}\right\| &\le |\lambda||\beta/4+it|e^{4|\beta/4+it|\cdot\|h\|_\infty}\max_i\|[V,\omega_i]\|\\ &\le c_2|\lambda|\cdot|\beta/4+it|\cdot e^{c_3|\beta/4+it|}\,,\end{aligned}\tag{B2}$$

which is independent of the system size when we assume that V contains only terms with even numbers of Majorana fermions, as is required for physical Hamiltonians, and has exponentially decaying interactions. Here the second to last inequality follows from submultiplicativity of ℓ_∞ norm (which is given by the maximal absolute row sum of the matrix). Finally, we get that

$$\|\sigma_\beta^{-1/4}L_a\sigma_\beta^{1/4} - \sigma_{\beta,0}^{-1/4}L_a^0\sigma_{\beta,0}^{1/4}\| \le \int_{-\infty}^{\infty}|f^a(t)|c_2|\lambda|\cdot|\beta/4+it|\cdot e^{c_3|\beta/4+it|}\mathrm{d}t = c_1|\lambda|$$

for some constant c_1 independent of the system size. Hence we can bound the dissipative parts of the parent Hamiltonian like

$$\begin{aligned}\left\|\sigma_\beta^{-1/4}L_a\sigma_\beta^{1/4}\otimes\overline{\sigma_\beta^{-1/4}L_a\sigma_\beta^{1/4}} - \sigma_{\beta,0}^{-1/4}L_a^0\sigma_{\beta,0}^{1/4}\otimes\overline{\sigma_{\beta,0}^{-1/4}L_a^0\sigma_{\beta,0}^{1/4}}\right\| &\le 2c_1|\lambda|\,,\\ \left\|\sigma_\beta^{-1/4}L_a^\dagger L_a\sigma_\beta^{1/4}\otimes I - \sigma_{\beta,0}^{-1/4}L_a^{0\dagger}L_a^0\sigma_{\beta,0}^{1/4}\otimes I\right\| &\le 2c_1|\lambda|\,,\\ \left\|I\otimes\overline{\sigma_\beta^{1/4}L_a^\dagger L_a\sigma_\beta^{-1/4}} - I\otimes\overline{\sigma_{\beta,0}^{1/4}L_a^{0\dagger}L_a^0\sigma_{\beta,0}^{-1/4}}\right\| &\le 2c_1|\lambda|\,.\end{aligned}$$

Now looking at the coherent term, we shall split it up into quasi-local contributions $G = \sum_a G_a$ with $G_a = \int_{-\infty}^{\infty} g(t)e^{iHt}(L_a^\dagger L_a)e^{-iHt}\,\mathrm{d}t$. Then we similarly need to bound $\|\sigma_\beta^{-1/4}G_a\sigma_\beta^{1/4} - \sigma_{\beta,0}^{-1/4}G_a^0\sigma_{\beta,0}^{1/4}\|$:

$$\begin{aligned}&\|\sigma_\beta^{-1/4}G_a\sigma_\beta^{1/4} - \sigma_{\beta,0}^{-1/4}G_a^0\sigma_{\beta,0}^{1/4}\|\\ &\quad\le\int_{-\infty}^{\infty}|g(t)|\left(\|L_a^\dagger L_a - L_a^{0\dagger}L_a^0\| + \left\|e^{H(\beta/4+it)}L_a^{0\dagger}L_a^0e^{-H(\beta/4+it)} - e^{H_0(\beta/4+it)}L_a^{0\dagger}L_a^0e^{-H_0(\beta/4+it)}\right\|\right)\mathrm{d}t\,.\end{aligned}$$

Here we can again use Lemma D.1 to bound

$$\left\|e^{H(\beta/4+it)}L_a^{0\dagger}L_a^0e^{-H(\beta/4+it)}-e^{H_0(\beta/4+it)}L_a^{0\dagger}L_a^0e^{-H_0(\beta/4+it)}\right\| \\ \leq |\lambda||\beta/4+it|\max_{s\in[0,1]}\left\|\left[V,e^{s(\beta/4+it)H_0}L_a^{0\dagger}L_a^0e^{-s(\beta/4+it)H_0}\right]\right\|.$$

Using the exact solution $L_a^0=\sum_i \hat{f}(-4h)_{ai}\omega_i$, we can upper bound this further like

$$\max_{s\in[0,1]}\|[V,e^{s(\beta/4+it)H_0}L_a^{0\dagger}L_a^0e^{-s(\beta/4+it)H_0}]\|\leq 2\|\hat{f}(-4h)\|_\infty^2\cdot e^{\beta\|h\|_\infty}\cdot w_h(t)\cdot\max_k\|[V,\omega_k]\| \\ \leq 2c_2e^{c_3\beta/4}\cdot w_h(t)\cdot\|\hat{f}(-4h)\|_\infty^2\,,$$

where $w_h(t)$ is system-size-independent function growing subexponentially in t (as discussed in Section E). Note that we have $\|\hat{f}(-4h)\|_\infty\leq e^{2\beta^2\|h\|_\infty^2+\beta\|h\|_\infty}$ due to submultiplicativity of the ℓ_∞ norm, and so $\|h\|_\infty=\mathcal{O}(1)$ ensures that $\|\hat{f}(-4h)\|_\infty=\mathcal{O}(1)$. Observe that the previous argument for bounding the conjugated expression $\left\|\sigma_\beta^{-1/4}L_a^\dagger L_a\sigma_\beta^{1/4}-\sigma_{\beta,0}^{-1/4}L_a^{0\dagger}L_a^0\sigma_{\beta,0}^{1/4}\right\|$ also shows that $\left\|L_a^\dagger L_a-L_a^{0\dagger}L_a^0\right\|\leq c_4|\lambda|$. Finally, this means that

$$\|\sigma_\beta^{-1/4}G_a\sigma_\beta^{1/4}-\sigma_{\beta,0}^{-1/4}G_a^0\sigma_{\beta,0}^{1/4}\|\leq\int_{-\infty}^{\infty}|g(t)|\cdot(c_4|\lambda|+|\lambda||\beta/4+it|c_5w_h(t))\ \mathrm{d}t=c_6|\lambda|,$$

where the convergence is ensured by the decay bounds of $g(t)$ obtained in [22, Lemma 30].

This proves that the strength of the perturbation of the parent Hamiltonian (in the vectorised picture) is upper bounded by a constant multiple of the strength of the perturbation of the system's Hamiltonian, uniformly in system size, i.e. that $\mathcal{V}_a$, where $\mathcal{V}=\sum_{a\in\mathcal{A}}\mathcal{V}_a$, is upper bounded like $\|\mathcal{V}_a\|\leq c|\lambda|$. To match the formulation of Definition B.4, we need to express $\mathcal{V}_a$ as a telescoping sum like $\mathcal{V}_a=\mathcal{V}_a^{(0)}+\sum_{r=1}^\infty\mathcal{V}_a^{(r)}-\mathcal{V}_a^{(r-1)}$, where $\mathcal{V}_a^{(r)}$ is a truncation of $\mathcal{V}_a$ to the ball $B_r(a)$ of radius r centred at a. This truncation then amounts to replacing H by a truncated version $H_{B_r(a)}$ in all the time-evolved formulae of the involved operators. The argument for bounding $\mathcal{V}_a$ directly translates to a bound on $\mathcal{V}_a^{(r)}$ and hence on $\varepsilon_a^{(r)}=\mathcal{V}_a^{(r)}-\mathcal{V}_a^{(r-1)}$. The quasi-locality of the parent Hamiltonians $\mathcal{H}$ and $\mathcal{H}_0$, and hence that of $\mathcal{V}$, was shown in Proposition 4, and stems from the Lieb-Robinson argument in [22, Proposition 20] proving quasi-locality at any temperature. As these properties are independent, they show together that $\|\varepsilon_a^{(r)}\|\leq c|\lambda|e^{-\mu r}$, and so $\mathcal{V}=\sum_{a\in\mathcal{A}}\sum_{r\geq0}\varepsilon_a^{(r)}$ has $(c|\lambda|,\mu)$-decay (where $\varepsilon_a^{(0)}\equiv\mathcal{V}_a^{(0)}$). □

In Lemma E.1, we also present a slightly weaker notion of this result, with the strength bounded by $|\lambda|^\alpha$ for an arbitrary constant $\alpha<1$ for small enough $|\lambda|$, which works for general Hamiltonians.

Theorem B.9. *Under the assumptions of Lemmas B.7 and B.8, at any inverse temperature β, there exist positive constants $\lambda_{\max}$ and d, such that the Lindbladian $\mathcal{L}^\dagger$ corresponding to the perturbed fermionic Hamiltonian $H=H_0+\lambda V$ has a spectral gap Δ lower bounded by $\Delta_0-d|\lambda|$ for any $|\lambda|\leq\lambda_{\max}$, where the unperturbed gap Δ_0 is specified in (B1); independent of system size.*

Proof. We want to bound the gap of $\mathcal{L}^\dagger$, and we wish to use the results about stability of gaps of free fermionic systems under perturbation. Hence we will consider the similarity transformation that will take $\mathcal{L}^\dagger$ to $\mathcal{H}=\mathcal{H}_0+\mathcal{V}$, where $\mathcal{H}_0$ is a Hermitian free fermionic Hamiltonian. As we have already shown, $\mathcal{H}_0$ has a gap Δ_0 and $[J,\nu]$-decay, while $\mathcal{V}$ has (K,μ)-decay, and hence by the stability Theorem B.6 there exist constants $K_{\max}$ and d_1, s.t. for all $K\leq K_{\max}$, the gap of the perturbed parent Hamiltonian, and hence the gap of the Lindbladian, is lower bounded like $\Delta\geq\Delta_0-d_1K$. But we also know that $K\leq c|\lambda|$, and hence there exists $\lambda_{\max}=\frac{K_{\max}}{c}$ such that whenever $|\lambda|\leq\lambda_{\max}$, we also have $K\leq K_{\max}$ and $\Delta\geq\Delta_0-d_1c|\lambda|$. □

While other filter functions (potentially in combination with other jump operators) might work significantly better in practice (see Section C), here we required superexponential decay of the filter function $f(t)$ in the time domain, ensuring the locality of the parent Hamiltonians for systems with exponentially decaying correlations, and the convergence of the integrals appearing in the particular bounds of the strength of the Lindbladian perturbation we use here — this lead us to use the Gaussian filter.

3. Stability of the Lindbladian Gap Under Perturbations of the Atomic Limit

In this section, we investigate the so-called atomic limit — where interactions among different sites are absent — and its perturbations. The atomic limit of the spinful Fermi-Hubbard Hamiltonian corresponds to setting $t=0$ in the Hamiltonian of Eq. (A11):

$$H_{\text{atomic}}=U\sum_{i=1}^n N_{i,\uparrow}N_{i,\downarrow}\,.$$

The following discussion can be easily generalised to any Hamiltonians that are separable in the lattice sites but for simplicity of exposition we will discuss here only the Fermi-Hubbard model at $t = 0$. H_{atomic} is trivially solvable and its eigenstates are given by electrons localized at the lattice sites. We will now show that if we choose local fermionic jump operators, the Lindbladian and parent Hamiltonian associated to H_{atomic} are also separable and we can compute exactly their spectrum.

Proposition B.10. *The Lindbladian $\mathcal{L}^{\dagger}_{\text{atomic}}$ corresponding to the Hamiltonian H_{atomic} with the set of jump operators $\{\omega_a\}_{a=1}^{2n}$ and Gaussian filter function is gapped for any $\beta \geq 0$ and $U \in \mathbb{R}$.*

Proof. For each $i \in \{1, \ldots, n\}$, $\alpha \in \{\uparrow, \downarrow\}$ we define the Lindblad operators and the operators $\tilde{L}$ associated to creation and annihilation operators:

$$
\begin{aligned}
L_{i,\alpha,-} &= \int_{-\infty}^{+\infty} f(t) e^{itH} a_{i,\alpha} e^{-itH} = \int_{-\infty}^{+\infty} f(t) e^{-itUN_{i,\bar{\alpha}}} a_{i,\alpha} = \hat{f}(-UN_{i,\bar{\alpha}}) a_{i,\alpha} \\
L_{i,\alpha,+} &= \int_{-\infty}^{+\infty} f(t) e^{itH} a^{\dagger}_{i,\alpha} e^{-itH} = \hat{f}(+UN_{i,\bar{\alpha}}) a^{\dagger}_{i,\alpha} \,, \\
\tilde{L}_{i,\alpha,-} &= \int_{-\infty}^{+\infty} f(t) e^{(\beta/4+it)H} a_{i,\alpha} e^{-(\beta/4+it)H} = q_-(N_{i,\bar{\alpha}}) a_{i,\alpha} \,, \\
\tilde{L}_{i,\alpha,+} &= \int_{-\infty}^{+\infty} f(t) e^{(\beta/4+it)H} a^{\dagger}_{i,\alpha} e^{-(\beta/4+it)H} = q_+(N_{i,\bar{\alpha}}) a^{\dagger}_{i,\alpha} \,,
\end{aligned}
$$

where $\bar{\alpha}$ is the opposite direction of the spin α, we used $[N, a] = -a$, $[N, a^{\dagger}] = a^{\dagger}$ to compute the time evolution of the oscillators, and defined

$$
q_{\pm}(x) \equiv q(\pm Ux) \,.
$$

Recall that the function q is related to the filter function as in (A2) and $\overline{q_+(\nu)} = q_-(\nu)$. Note that

$$
\tilde{L}^{\dagger}_{i,\alpha,+} = \overline{q_+(N_{i,\bar{\alpha}})} a_{i,\alpha} = \tilde{L}_{i,\alpha,-} \,.
$$

Now we take the self-adjoint Majorana operators as jump operators:

$$
\omega_{i,\alpha,0} = \frac{1}{\sqrt{2}}(a_{i,\alpha} + a^{\dagger}_{i,\alpha}) \,, \quad \omega_{i,\alpha,1} = \frac{-i}{\sqrt{2}}(a_{i,\alpha} - a^{\dagger}_{i,\alpha}) \,,
$$

and the corresponding self-adjoint operators:

$$
\tilde{L}_{i,\alpha,0} = \frac{1}{\sqrt{2}}(\tilde{L}_{i,\alpha,-} + \tilde{L}_{i,\alpha,+}) = \tilde{L}^{\dagger}_{i,\alpha,0} \,, \quad \tilde{L}_{i,\alpha,1} = \frac{-i}{\sqrt{2}}(\tilde{L}_{i,\alpha,-} - \tilde{L}_{i,\alpha,+}) = \tilde{L}^{\dagger}_{i,\alpha,1} \,.
$$

For the operators $L^{\dagger}_a L_a$, we have

$$
L^{\dagger}_{i,\alpha,-} L_{i,\alpha,-} = |\hat{f}(-UN_{i,\bar{\alpha}})|^2 N_{i,\alpha} \,, \quad L^{\dagger}_{i,\alpha,+} L_{i,\alpha,+} = |\hat{f}(+UN_{i,\bar{\alpha}})|^2 (1 - N_{i,\alpha}) \,, \quad L^{\dagger}_{i,\alpha,+} L_{i,\alpha,-} = L^{\dagger}_{i,\alpha,-} L_{i,\alpha,+} = 0 \,.
$$

Note that $[H, L^{\dagger}_{i,\alpha,z} L_{i,\alpha,z}] = 0$ for all indices so that only the $\nu = 0$ component of $(L^{\dagger}_{i,\alpha,z} L_{i,\alpha,z})_{\nu}$ is non-zero and thus $G = 0$. Further, denoted

$$
F(N_{i,\alpha}, N_{i,\bar{\alpha}}) := \frac{1}{2}(L^{\dagger}_{i,\alpha,+} L_{i,\alpha,+} + L^{\dagger}_{i,\alpha,-} L_{i,\alpha,-}) = \frac{1}{2}(|\hat{f}(+UN_{i,\bar{\alpha}})|^2 (1 - N_{i,\alpha}) + |\hat{f}(-UN_{i,\bar{\alpha}})|^2 N_{i,\alpha}) \,, \tag{B3}
$$

we have

$$
\begin{aligned}
\tilde{M}_{i,\alpha,0} &= L^{\dagger}_{i,\alpha,0} L_{i,\alpha,0} = \frac{1}{2}(L^{\dagger}_{i,\alpha,+} + L^{\dagger}_{i,\alpha,-})(L_{i,\alpha,+} + L_{i,\alpha,-}) = F(N_{i,\alpha}, N_{i,\bar{\alpha}}) \\
\tilde{M}_{i,\alpha,1} &= L^{\dagger}_{i,\alpha,1} L_{i,\alpha,1} = \frac{1}{2}(L^{\dagger}_{i,\alpha,+} - L^{\dagger}_{i,\alpha,-})(L_{i,\alpha,+} - L_{i,\alpha,-}) = F(N_{i,\alpha}, N_{i,\bar{\alpha}}) \,.
\end{aligned}
$$

Then the Lindbladian and parent Hamiltonian are

$$
\begin{aligned}
\mathcal{L} &= \sum_{i=1}^{n} \sum_{\alpha \in \{\uparrow,\downarrow\}} \sum_{z=0}^{1} \mathcal{L}_{i,\alpha,z} \,, \quad \mathcal{L}_{i,\alpha,z}(\rho) = L_{i,\alpha,z} \rho L^{\dagger}_{i,\alpha,z} - \frac{1}{2}\{F(N_{i,\alpha}, N_{i,\bar{\alpha}}), \rho\} \\
\mathcal{H} &= \sum_{i=1}^{n} \sum_{\alpha \in \{\uparrow,\downarrow\}} \sum_{z=0}^{1} \mathcal{H}_{i,\alpha,z} \,, \quad \mathcal{H}_{i,\alpha,z}(\rho) = \widetilde{L}_{i,\alpha,z} \rho \widetilde{L}_{i,\alpha,z} - \frac{1}{2}\{F(N_{i,\alpha}, N_{i,\bar{\alpha}}), \rho\} \,.
\end{aligned}
$$

They are separable and therefore exactly solvable. The eigenstates of $\mathcal{H}$ are of the form

$$\rho = \bigotimes_{i=1}^{n} \rho_i \,,$$

where ρ_i is a solution to the reduced eigenproblem:

$$\sum_{z=0}^{1}(\tilde{L}_{\uparrow,z}\sigma\tilde{L}_{\uparrow,z} + \tilde{L}_{\downarrow,z}\sigma\tilde{L}_{\downarrow,z}) - \{F(N_\uparrow, N_\downarrow) + F(N_\downarrow, N_\uparrow), \sigma\} = e\sigma \,, \tag{B4}$$

where we suppress the i index for notational simplicity. To diagonalise this, we use a Jordan-Wigner transformation where we identify $\uparrow\equiv 1$, $\downarrow\equiv 2$:

$$N_\alpha = \frac{1}{2}(\mathbf{1} + Z_\alpha)\,, \quad a_1 = \sigma_1^- \,, \quad a_1^\dagger = \sigma_1^+ \,, \quad a_2 = Z_1\sigma_2^- \,, \quad a_2^\dagger = Z_1\sigma_1^+ \,,$$

so that

$$\begin{aligned}
\widetilde{L}_{\uparrow,0} &= \frac{1}{\sqrt{2}}(q_-(N_2)a_1 + q_+(N_2)a_1^\dagger) = \frac{1}{\sqrt{2}}(q_-(N_2)\sigma_1^- + q_+(N_2)\sigma_1^+) \\
\widetilde{L}_{\downarrow,0} &= \frac{1}{\sqrt{2}}(q_-(N_1)a_2 + q_+(N_1)a_2^\dagger) = \frac{1}{\sqrt{2}}(q_-(N_1)Z_1\sigma_2^- + q_+(N_1)Z_1\sigma_2^+) \\
\widetilde{L}_{\uparrow,1} &= \frac{-i}{\sqrt{2}}(q_-(N_2)a_1 - q_+(N_2)a_1^\dagger) = \frac{-i}{\sqrt{2}}(q_-(N_2)\sigma_1^- - q_+(N_2)\sigma_1^+) \\
\widetilde{L}_{\downarrow,1} &= \frac{-i}{\sqrt{2}}(q_-(N_1)a_2 - q_+(N_1)a_2^\dagger) = \frac{-i}{\sqrt{2}}(q_-(N_1)Z_1\sigma_2^- - q_+(N_1)Z_1\sigma_2^+)\,.
\end{aligned}$$

The stationary state of the Lindbladian is σ_β and its eigenvalue is 0. The gap then corresponds to the highest non-zero eigenvalue of (B4) — recall that the spectrum is non-positive. It can be easily computed by diagonalising numerically a 16×16 matrix corresponding to the vectorised reduced Hamiltonian. We plot the result in Supplementary Figure 1. We see that the gap is non-zero and goes to 0 as $|U| \to \infty$. This result holds for any system size.

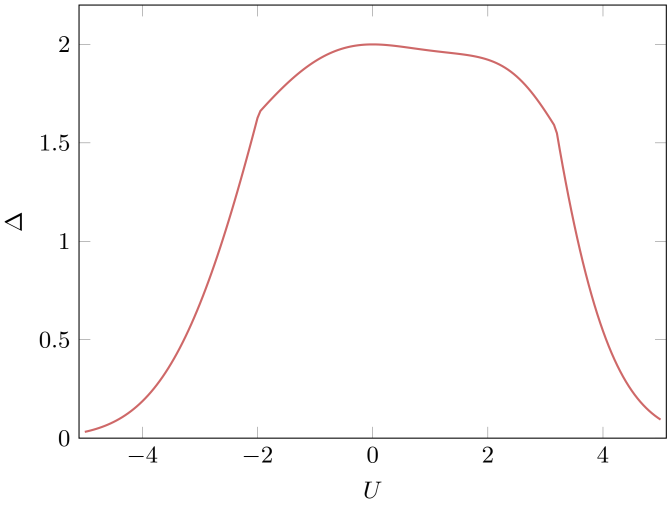

Supplementary Figure 1: Gap of the Lindbladian for the spinful Fermi-Hubbard model at $t = 0$. We set $\beta = 1$, fix the Gaussian filter and vary U.

□

The gap, however, closes for zero temperature as the next proposition shows.

Proposition B.11. *The gap of the Lindbladian $\mathcal{L}^\dagger_{\text{atomic}}$ corresponding to the atomic Hamiltonian H_{atomic} with the set of jump operators $\{\omega_a\}_{a=1}^{2n}$ and Gaussian filter function closes as $\beta \to \infty$.*

Proof. We shall show that $\mathbf{1}$ becomes degenerate with σ_β as $\beta \to \infty$ for any system size. We shall use here results and notations from the proof of Prop. B.10. Since $G = 0$, we have

$$\mathcal{L}^\dagger_{\text{atomic}}(\mathbf{1}) = \sum_{i=1}^{n} \sum_{\alpha\in\{\uparrow,\downarrow\}} \sum_{z=0}^{1} [L_{i,\alpha,z}, L^\dagger_{i,\alpha,z}]$$

We know from equation (B3) that

$$L^\dagger_{i,\alpha,+}L_{i,\alpha,+} + L^\dagger_{i,\alpha,-}L_{i,\alpha,-} = |\hat f(+UN_{i,\bar\alpha})|^2(1-N_{i,\alpha}) + |\hat f(-UN_{i,\bar\alpha})|^2 N_{i,\alpha}$$

and we also have

$$L_{i,\alpha,+}L^\dagger_{i,\alpha,+} + L_{i,\alpha,-}L^\dagger_{i,\alpha,-} = |\hat f(+UN_{i,\bar\alpha})|^2 N_{i,\alpha} + |\hat f(-UN_{i,\bar\alpha})|^2(1-N_{i,\alpha})$$

These two expressions cancel so that $\mathcal{L}^\dagger_{\text{atomic}}(\mathbf{1}) = 0$ if $\hat f$ is even. From (A7) we see that for large β the Gaussian filter is indeed even, which proves the proposition. □

Next we discuss the robustness of the gap of $\mathcal{L}^\dagger_{\text{atomic}}$ under weak interactions among sites. This follows by realising that the associated parent Hamiltonian is geometrically local and frustration-free — that is, it is a sum of local terms and the ground state is an eigenstate of each of them — and that the perturbation to the parent Hamiltonian is quasi-local. We can then use results about the stability of the gap for frustration-free Hamiltonians, such as [46, 68, 69] and also the older works on quantum perturbations of classical systems [70, 71], to prove the stability of the gap of the perturbed Lindbladian. In particular, we can prove the following.

Theorem B.12. *Consider the interacting fermionic Hamiltonian $H = H_{\text{atomic}} + \lambda V$ and assume that V has interactions that decay at least exponentially. Then at any inverse temperature β, there exist positive constants $\lambda_{\max}$ and d, such that the Lindbladian $\mathcal{L}^\dagger$ corresponding to the perturbed Hamiltonian H has a spectral gap Δ lower bounded by $\Delta_{\text{atomic}} - d|\lambda|^\alpha$ for any $|\lambda| \le \lambda_{\max}$ and arbitrary positive constant $\alpha < 1$, where Δ_{atomic} is the gap of the Lindbladian $\mathcal{L}^\dagger_{\text{atomic}}$ of Prop. B.10.*

Proof. Let us denote by $e_\ell \le \cdots \le e_1 < e_0 \equiv 0$ the set of eigenvalues of the single site parent Hamiltonian in the atomic limit, equation (B4). Then $|e_1| > 0$ is the Lindbladian gap in the atomic limit. Now we consider the Hamiltonian

$$\mathcal{H}_0 = \sum_{i=1}^n \sum_{a=0}^\ell E_a(\mathcal{P}_{e_a})_i\,, \quad E_a = -\frac{e_a}{e_1}\,,$$

where $(\mathcal{P}_e)_i$ is the projector onto the eigenspace of e acting at site i. $\mathcal{H}_0$ is a rescaled and negated version of the parent Hamiltonian at $t=0$ such that $\mathcal{H}_0 \ge 0$. It has a non-degenerate ground state with eigenvalue 0 given by the Fermi-Hubbard thermal state at $t=0$ and gap 1. Now we denote by $\tilde{\mathcal{H}}(\lambda)$ minus the parent Hamiltonian associated to H, so that the spectrum is positive and the thermal state is the ground state. Then $\tilde{\mathcal{H}}(0) = e_1\mathcal{H}_0$ and we define

$$\mathcal{V} = \tilde{\mathcal{H}}(\lambda) - \tilde{\mathcal{H}}(0) = \tilde{\mathcal{H}}(\lambda) - e_1\mathcal{H}_0\,.$$

Next we will prove a gap for $\mathcal{H} = \mathcal{H}_0 + \mathcal{V}$ using the following result.

Lemma B.13 (Theorem 4 of [46])**.** *There exist constant J_0, c_1 depending only on $\tilde J, \tilde\mu, \mu, D$ such that the following holds for all $J \le J_0$. Let H_0 have $(\tilde J, \tilde\mu)$ decay and let V have (J,μ) decay, according to the notion of decay in Definition B.4. Assume $H_0 \ge H_{proj}$ for some H_{proj} which is a sum of commuting projectors and which obeys the topological quantum order conditions of [68]. Assume also $PH_0 = 0$ where P is the projector onto the ground state of H_{proj}. Then the spectral gap of $H_0 + V$ is at least $1 - c_1 J - \delta$, for some δ bounded by J times a quantity decaying faster than any power of L.*

We are going to use this result with H_0 identified with $\mathcal{H}_0$. This means that $\tilde\mu = \infty$. Then we define a Hamiltonian $\mathcal{H}_{\text{proj}}$ which we identify with H_{proj} by:

$$\mathcal{H}_{\text{proj}} = \sum_{i=1}^n Q_i\,, \quad Q_i = 1 - P_i\,, \quad P_i \equiv (\mathcal{P}_0)_i\,.$$

Let us denote $P_A = \prod_{i\in A} P_i$ for a set of sites A, and the projector onto the ground state of $\mathcal{H}_{\text{proj}}$ by

$$P = \prod_{i=1}^n (1 - Q_i) = \prod_{i=1}^n P_i\,.$$

Note that the ground state of $\mathcal{H}_{\text{proj}}$ is non-degenerate and P_A is a rank one projector for any set of sites A. We have the following properties:

(i) $\mathcal{H}_0 - \mathcal{H}_{\text{proj}} \ge 0$. Indeed $P\mathcal{H}_0 = P\mathcal{H}_{\text{proj}} = 0$ so that $\mathcal{H}_0, \mathcal{H}_{\text{proj}}$ have the same ground state. Also they have the same gap 1 and the other eigenvalues of $\mathcal{H}_0$ are greater or equal to those of $\mathcal{H}_{\text{proj}}$ since $[\mathcal{H}_0, \mathcal{H}_{\text{proj}}] = 0$ and $E_a \ge 1$ for $a > 1$.

(ii) $\mathcal{H}_{\text{proj}}$ is the sum of local commuting projectors.

(iii) $\mathcal{H}_{\text{proj}}$ satisfies the topological quantum order conditions [68]

(a) TQO-1: if O_A is supported on A then

$$PO_AP = P_AO_AP_AP_{A^\perp} = \text{Tr}(P_AO_A)P = cP$$

where $A^\perp = \Lambda \setminus A$. This happens for any A, so $L^* = L$, the system size.

(b) TQO-2: If $PO_A = P_AO_AP_{A^\perp} = 0$, then $P_AO_A = 0$, and so $P_BO_A = 0$ if B includes A.

Finally we identify V with $\mathcal{V}$ and the last hypothesis of Lemma B.13 to verify the (J,μ) decay of $\mathcal{V}$. This follows from Lemma E.1 which shows that $J \le c|\lambda|^\alpha$ for any $0 < \alpha < 1$. Lemma B.13 then implies that $\mathcal{H}$ is gapped for all $|\lambda|^\alpha \le J_0/c$ and that the gap is $1 - \mathcal{O}(|\lambda|^\alpha)$. This in turns implies a gap $|e_1| - \mathcal{O}(|\lambda|^\alpha)$ for $\tilde{\mathcal{H}}(\lambda) = e_1(\mathcal{H}_0 + e_1^{-1}\mathcal{V})$ and thus for the Lindbladian $\mathcal{L}^\dagger$ for all $|\lambda| \le \lambda_{\max}$. Here $\lambda_{\max} = (|e_1|J_0/c)^{1/\alpha}$ since the strength of the perturbation in $\tilde{\mathcal{H}}(\lambda)/e_1$ is $J \le c|e_1^{-1}||\lambda|^\alpha$. □

Note that Theorem 3 in particular implies a gap for the Lindbladian associated with the spinful Fermi-Hubbard model for weak interactions around the atomic limit by identifying λ with t. Note also that similar conclusions about perturbations of the atomic limit can be drawn with Pauli jump operators that we discuss in Section C 2. This is because the atomic Lindbladian and parent Hamiltonian remain separable also in that case.

We end this section with the remark that the spinless Fermi-Hubbard model at $t = 0$ is not separable, see Eq. (A12). Thus it does not reduce to an atomic limit and the results of this section do not apply to perturbations of the $t = 0$ limit in the spinless case. However, commutativity of the Hamiltonian implies that the Lindbladian and the parent Hamiltonians for the $t = 0$ spinless Fermi-Hubbard model are strictly local. We leave investigations of this model for future work.

4. Efficient Quantum Gibbs Sampler

In this section, we shall finally explain how a constant lower bound on the spectral gap of the Lindbladian translates to the bound on the mixing time and hence the overall algorithmic complexity of the Gibbs state preparation, proving its efficiency.

Corollary B.13.1. *The mixing time t_{mix} of the Lindbladian $\mathcal{L}^\dagger$ can be then bounded like*

$$t_{\text{mix}} \le \frac{\log\left(\frac{2}{\epsilon}\|\sigma_\beta^{-1/2}\|\right)}{\Delta} = \frac{\mathcal{O}(\beta\|H\| + \log(1/\epsilon))}{\Delta} = \mathcal{O}(n + \log(1/\epsilon)).$$

Proof. As per [3, Proposition E.4], we can bound

$$\left\|e^{\mathcal{L}^\dagger t}[\rho_1 - \rho_2]\right\|_{\text{Tr}} \le e^{-\Delta(\mathcal{L}^\dagger)t}\left\|\sigma_\beta^{-1/2}\right\| \|\rho_1 - \rho_2\|_{\text{Tr}}$$

using the Hölder's inequality; hence by taking $\rho_2 = \sigma_\beta$ to be the fixed point of the evolution, we get that

$$\left\|e^{\mathcal{L}^\dagger t}[\rho_1] - \sigma_\beta\right\|_{\text{Tr}} \le e^{-\Delta(\mathcal{L}^\dagger)t}\left\|\sigma_\beta^{-1/2}\right\| \|\rho_1 - \sigma_\beta\|_{\text{Tr}} \le 2e^{-\Delta(\mathcal{L}^\dagger)t}\left\|\sigma_\beta^{-1/2}\right\| \overset{\text{set}}{\le} \epsilon\,.$$

The last inequality is then guaranteed whenever $t \ge \frac{\log\left(\frac{2}{\epsilon}\left\|\sigma_\beta^{-1/2}\right\|\right)}{\Delta(\mathcal{L}^\dagger)}$, from which we can deduce

$$t_{\text{mix}} \le \frac{\log\left(\frac{2}{\epsilon}\left\|\sigma_\beta^{-1/2}\right\|\right)}{\Delta(\mathcal{L}^\dagger)}\,.$$

The rest of the bound follows from the spectral gap being lower bounded by a constant and $\|H\| = \mathcal{O}(n)$. □

Corollary B.13.2. *The purified Gibbs state can be prepared on a quantum computer at any constant temperature via Hamiltonian simulation of the parent Hamiltonian in*

$$\widetilde{\mathcal{O}}(n^3\,\text{polylog}(1/\epsilon))$$

time complexity using $\mathcal{O}(n)$ qubits, where ϵ is the desired precision in trace norm and $\widetilde{\mathcal{O}}$ notation absorbs subdominant polylogarithmic terms.

Proof. This follows from equation (A9) by using the upper bound on the mixing time, and the fact that $|\mathcal{A}| = \mathcal{O}(n)$ using the Majorana jump operators. □

5. Calculating Partition Functions

As a possible application of the efficient Gibbs state preparation, we adapt the strategy from [31] for calculating partition functions $Z_\beta(\lambda_i) = \mathrm{Tr}\big(e^{-\beta H(\lambda_i)}\big)$ to the case of interacting fermionic systems, where we have denoted $H(\lambda_i) = H_0 + \lambda_i V$. We remark that since we are assuming β to be constant (although arbitrarily large), the method in [31] based on cooling the partition function from infinite temperature is directly applicable, as their restriction to high temperatures stems only from the Gibbs state preparation. However, since we can consider β to be large and the coupling strength λ to be small, it might be more efficient in practice to consider systematically increasing the coupling strength rather than decreasing the temperature. Note that we can calculate the non-interacting partition function explicitly as

$$Z_\beta(0) = \prod_{i=1}^{n} 2\cosh(2\beta\epsilon_i)\,,$$

where the product is taken only over one $\epsilon_i \in \mathrm{spec}(h)$ from each symplectic pair $\pm\epsilon_i$. By measuring the observable $e^{\beta H(\lambda_i)}e^{-\beta H(\lambda_{i+1})}$ in the state $\sigma_\beta(\lambda_i) = \frac{e^{-\beta H(\lambda_i)}}{Z_\beta(\lambda_i)}$, we would obtain the ratio $\frac{Z_\beta(\lambda_{i+1})}{Z_\beta(\lambda_i)}$. Preparing this observable and the Gibbs state will require access to block encodings of H_0 and V, from which we get a block encoding for $H(\lambda_i)$ via LCU, and hence block encoding for the observable and the Hamiltonian simulation via QSVT. By choosing a schedule $0 = t_1 \le t_2 \le \cdots \le t_{l-1} \le t_l = |\lambda|$ and denoting $\lambda_i = t_i\frac{\lambda}{|\lambda|}$, we can calculate $Z_\beta(\lambda)$ as a telescoping product

$$Z_\beta(\lambda) = Z_\beta(0)\prod_{i=1}^{l-1}\frac{Z_\beta(\lambda_{i+1})}{Z_\beta(\lambda_i)} = Z_\beta(0)\prod_{i=1}^{l-1}\mathrm{Tr}\Big(e^{\beta H(\lambda_i)}e^{-\beta H(\lambda_{i+1})}\cdot\sigma_\beta(\lambda_i)\Big)\,.$$

Hence we can show the following adaptation of [31, Theorem 8]:

Corollary B.13.3. *For quasi-local interacting fermionic Hamiltonians $H(\lambda) = H_0 + \lambda V$, at any inverse temperature β, there exists a positive constant $\lambda_{\max}$ such that we can calculate an estimate to the partition function $Z_\beta(\lambda)$ up to a relative error ϵ with success probability at least $3/4$ for any $|\lambda| \le \lambda_{\max}$ in time complexity $\widetilde{\mathcal{O}}(n^5\epsilon^{-2})$.*

We refer to [31, Appendix C] for the details of these calculations, the gist of which lies in choosing the schedule such that $t_{i+1} - t_i = \Theta(n^{-1})$, and so $l = \Theta(n)$ as $\lambda = \Theta(1)$. Then we would prepare the Gibbs states $\sigma_\beta(\lambda_i)$ and measure the expectation values of the observables $e^{\beta H(\lambda_i)}e^{-\beta H(\lambda_{i+1})}$ for each $i \in [l-1]$ at least $\Theta(n\epsilon^{-2})$ times. Calculating the estimate for each $\frac{Z_\beta(\lambda_{i+1})}{Z_\beta(\lambda_i)}$ as the average over these measurements, and evaluating the estimate $\hat{Z}_\beta(\lambda)$ to the partition function using the telescoping product would hence ensure

$$\mathbb{P}\Big((1-\epsilon)Z_\beta(\lambda) \le \hat{Z}_\beta(\lambda) \le (1+\epsilon)Z_\beta(\lambda)\Big) \ge 3/4\,.$$

Appendix C: Numerical simulations

1. Analytically Bounded Regime

In this section, we investigate the Fermi-Hubbard model in the parameter range where Theorem 1 holds for the gap of the Lindbladian, i.e. where U is sufficiently small. Without loss of generality, we set $t = 1$ for all calculations where nothing to the contrary is explicitly stated.

Even though, as mentioned before, the one-dimensional Fermi-Hubbard model can be solved exactly using a Bethe-ansatz [50], we mostly focus our numerical analysis on this 1D setting. The main motivation for this is that we want to show finite-size scaling properties, which would demand too many classical resources for higher-dimensional settings. Still, where applicable, we also add small-scale 2D calculations on a $2 \times N$ grid.

In one dimension, the relevant Hamiltonian is that of a non-periodic fermionic chain, which in the spinless case has the free free fermionic part

$$H_0 = -t\sum_{i=1}^{n-1}(a_i^\dagger a_{i+1} + a_{i+1}^\dagger a_i) =: \sum_{i,j}\omega_i h_{ij}\omega_j\,. \tag{C1}$$

The spectrum of its single-particle Hamiltonian can be solved explicitly:

$$\mathrm{spec}(h) = 2\times\left\{\frac{t}{2}\cos\left(\pi\cdot\frac{k}{n+1}\right)\right\}_{k=1}^{n}\,.$$

Recall that the main Theorem 1 holds for Gaussian filter functions and Majorana jump operators. Choosing $\hat{f}(\nu) = e^{-(\beta\nu+1)^2/8+1/8}$ as the filter, we can use Eq. (B1) to determine the gap of the Lindbladian as

$$\begin{aligned} \Delta_0 &= 2e^{-4\beta^2\|h\|^2}\cosh(2\beta\|h\|) \\ &= 2e^{-\beta^2 t^2 \cos\left(\frac{\pi}{n+1}\right)^2}\cosh\left(\beta t \cos\left(\frac{\pi}{n+1}\right)\right) \\ &\geq 2e^{-\beta^2 t^2}\cosh(\beta t) =: \underline{\Delta}_0 \end{aligned} \tag{C2}$$

Supplementary Figure 2 shows the gap Δ of the Lindbladian for the spinless 1D Fermi-Hubbard model at $\beta = 3$ across various system sizes up to 11 sites. The analytical result from Eq. (C2), drawn in dashed grey (----), matches the markers of the numerical simulations at $U = 0$ (•). As U increases, Δ continuously deviates from this analytical result; initially shrinking the gap across the whole observed range, but for larger U only decreasing the gap for small system sizes, while seemingly saturating earlier and at larger values as the system size increases. This can be seen as a pointer that the actual asymptotic behaviour of this system might be even better than the analytical results suggest.

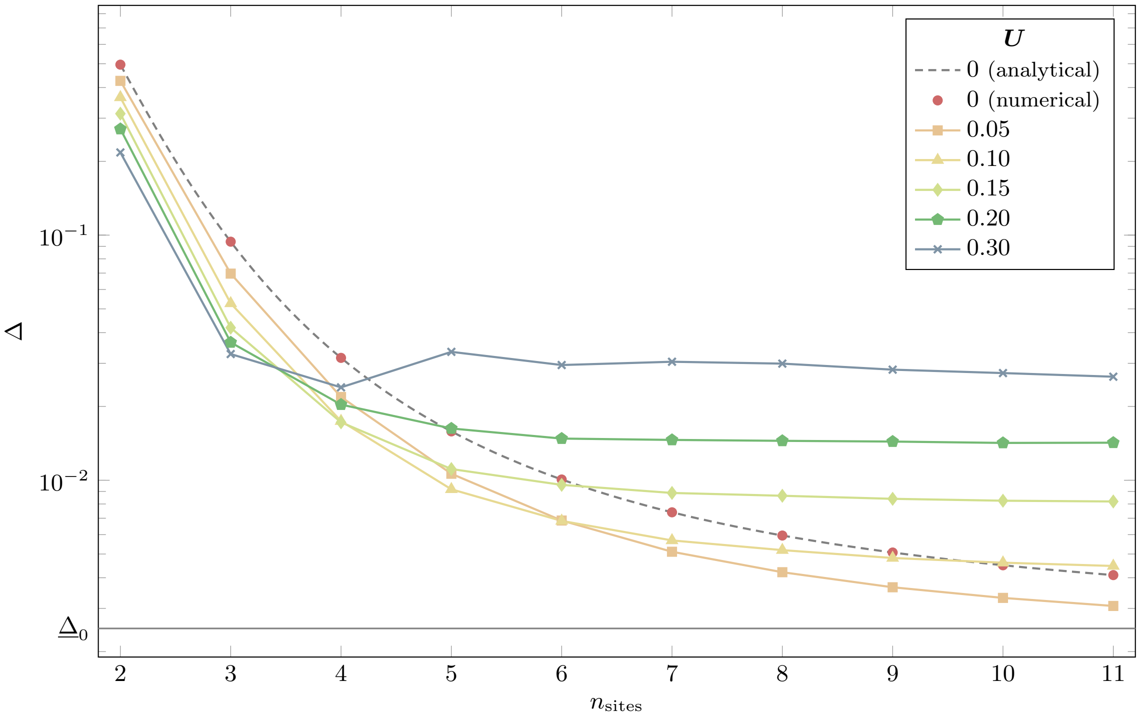

Supplementary Figure 2: Lindbladian gap Δ of the spinless 1D Fermi-Hubbard model at inverse temperature $\beta = 3$ for small interaction strengths U. The dashed grey line and red dots are analytically and numerically, respectively, derived results for $U = 0$, with the horizontal line at $\underline{\Delta}_0 := 2e^{-\beta^2 t^2}\cosh(\beta t)$ being the lower bound for this line for $n_{\text{sites}} \to \infty$.

For weak interactions, we can examine the analytical results more closely. For this particular fermionic system, where the strength of the perturbation is U, Theorem 1 takes the form $\Delta \geq \Delta_0 - d|U|$ as long as $|U| \leq U_{\max}$ with Δ being the gap of the perturbed system, Δ_0 the gap of the unperturbed system, some constant d, and a critical perturbation $U_{\max}$. It is important to stress that this inequality holds *independent of the system size.*

While it is difficult to numerically determine $U_{\max}$, we can get some idea about what d might be by looking at the derivative of Δ at $U = 0$. (Note that the gap Δ is not differentiable at $U = 0$, so we take the derivative in the positive direction 0^+ (repulsive Fermi-Hubbard model) and the negative direction 0^- (attractive FH-model) separately.) Supplementary Figure 3 shows a few such instances for different parameters of temperature, spinfulness, and sign of U. Because, as stated above, for a given β the constant d is independent of the system size, the theorem states that each line is bounded *from above* (lower is better). Indeed, for the considered temperatures the lines seem either decrease or tend to (almost) saturate even at the small system sizes shown in the plot.

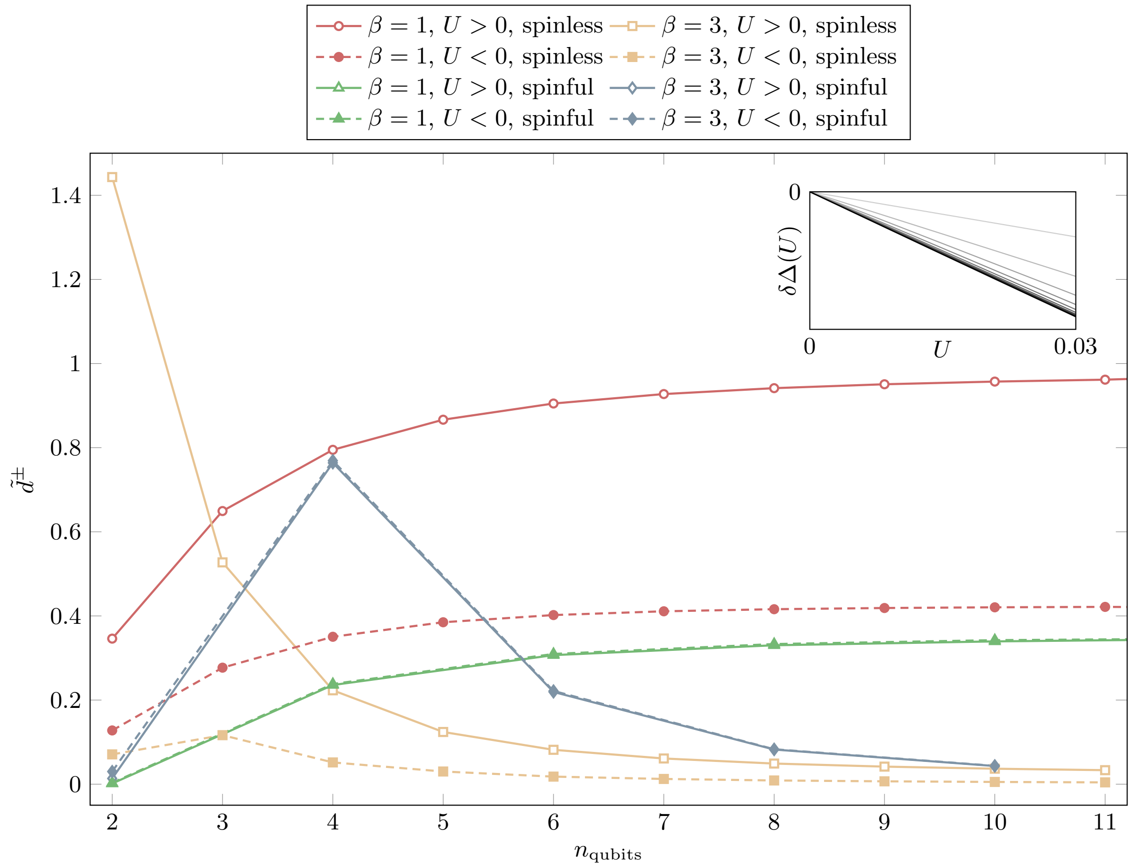

Supplementary Figure 3: Numerically evaluated slope of the gap $\tilde{d}^{+} = -\frac{\partial \Delta}{\partial U}\big|_{U=0^{+}}$ at positive U for the repulsive and $\tilde{d}^{-} = \frac{\partial \Delta}{\partial U}\big|_{U=0^{-}}$ at negative U for the attractive 1D Fermi-Hubbard model at different inverse temperatures β depending on the system size. Note that for the model with spin, the x-axis is the number of total sites when counting different spins separately, which matches the number of qubits required to represent the system. **Inset:** The deviation of the gap $\Delta(U)$ from the gap at $U = 0$, i.e. $\delta\Delta(U) \coloneqq \Delta(U) - \Delta(0)$ for the spinless Fermi-Hubbard model at $\beta = 1$. Darker colours correspond to more sites. The apparent clustering of lines towards some "slope bound" as they become darker is equivalent to the corresponding line in the main plot (—o—) approaching some upper bound.

2. Beyond Analytically Bounded Regime

So far — in the analytical as well as the numerical considerations — we have only used Gaussian filter functions and Majorana jump operators, as well as small perturbations. In this subsection, we extend our numerical results into regimes where the analytical guarantees of Theorem 1 may no longer hold. This will give some indication whether it seems reasonable that Gibbs states of the Fermi-Hubbard model can be prepared efficiently using the discussed algorithm. These small-scale insights might also inspire heuristics for which hyperparameters of the algorithm (filter functions, jump operators) can perform well even for larger system sizes. As before, we set $t = 1$ everywhere unless otherwise stated.

a. Strong coupling regime First, we maintain the Gaussian filter function and Majorana jump operators, but increase the interaction strength to much higher levels than before. Supplementary Figure 4 shows the gap Δ of the Lindbladian for several values of U. It seems that with this setup — at least for the 1D case and the specific temperatures shown — increasing the interaction strength tends to *shrink* the gap for high temperatures, but *grow* it for lower temperatures.

Overall, it seems that the strong coupling does not change the characteristics of Lindbladian gap for the worse. Even in the cases where the coupling shrinks the gap, the (limited) asymptotic behaviour looks very similar, saturating at a comparable rate as the (provably bounded) $U = 0$ case. In many other cases, the saturation of the bound seemingly arrives much earlier, and at a larger Δ than in the non-interacting case. Of course, these small-scale results must be treated with caution and in no way guarantee that the gap will stay open for arbitrarily large systems. But they nonetheless give some confidence that the Gibbs state preparation may also be efficient for larger systems with the investigated setup.

b. Metropolis filter function One problem that comes with using a Gaussian filter function is apparent in Eqs. (B1) and (C2), which is the dependence of the (unperturbed) Lindbladian gap Δ_0 on the inverse

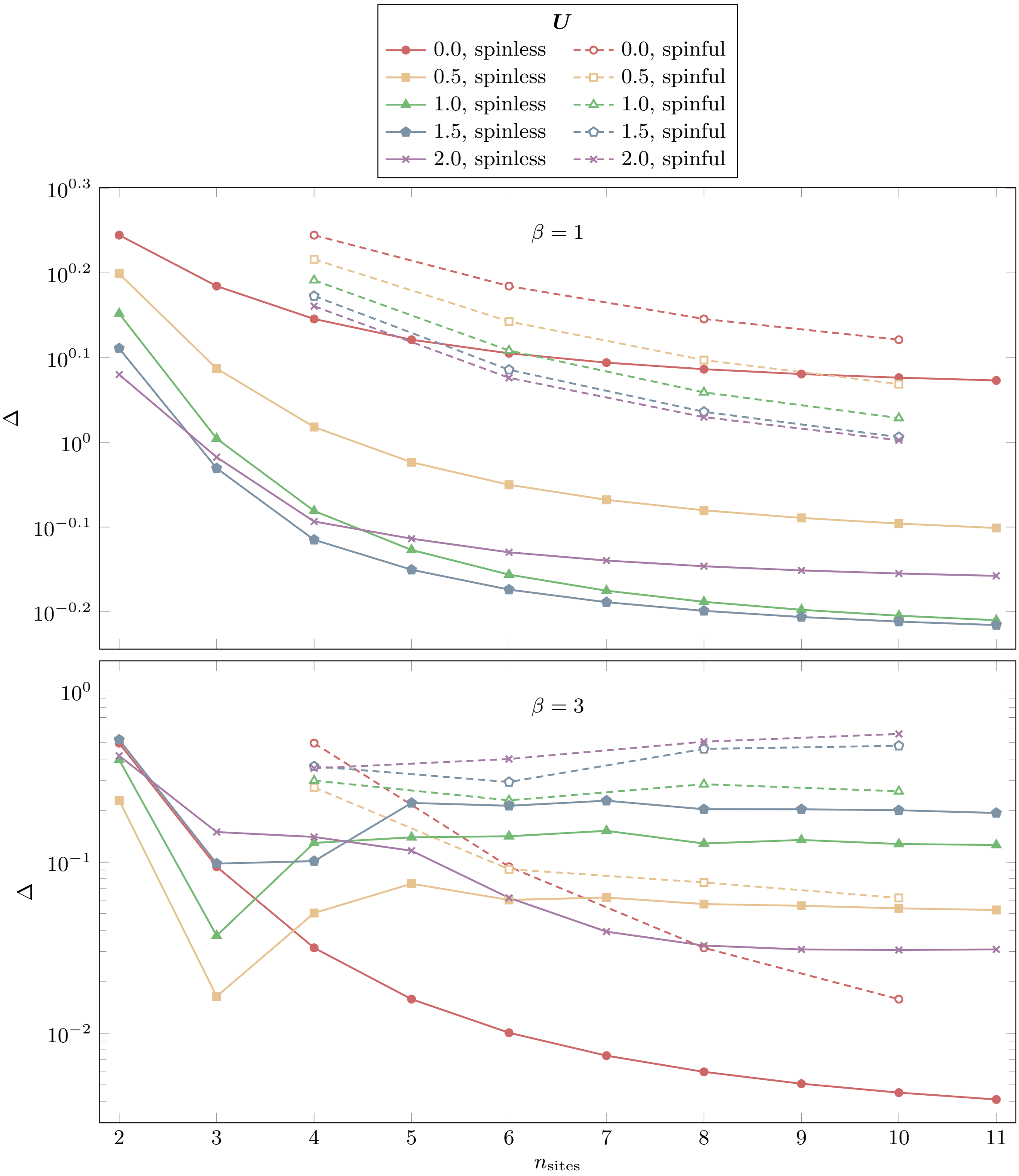

Supplementary Figure 4: Gap Δ of the Lindbladian for the spinless and spinful 1D Fermi-Hubbard model in the regime of strong interactions. The filter function remains Gaussian, the jump operators are single-site Majorana operators. In the spinful model, spin-up and spin-down are counted as separate sites, resulting in only even-numbered n_{sites}.

temperature β as $\Delta_0 \sim e^{-\beta^2}$. This means that while for high temperatures the gap might be substantial, it closes relatively rapidly upon cooling of the model. The authors of Reference [22] also note that using a Gaussian filter function can cause inefficiencies because the size of its support shrinks with β^{-1}, limiting the available transitions between energy eigenstates.

It therefore seems reasonable to investigate the behaviour of the gap Δ when using a different kind of filter function. We opt for a Metropolis-type filter mentioned in Eq. (A8), which is

$$\hat{f}^a(\nu) = e^{-\sqrt{1+\beta^2\nu^2}}\, w(\nu/S)\, e^{-\beta\nu/4}$$

where we now use $S = 10$.

The gap of the unperturbed Lindbladian is then

$$\begin{aligned}\Delta_0 &= 2 \cdot \min_i q(4\epsilon_i)^2 \cosh(2\beta\epsilon_i) \\ &= 2 \cdot \min_i e^{-2\sqrt{1+16\beta^2\epsilon_i^2}} w(4\epsilon_i/S)^2 \cosh(2\beta\epsilon_i) \\ &= 2e^{-2\sqrt{1+16\beta^2\|h\|^2}} w(4\|h\|/S)^2 \cosh(2\beta\|h\|)\,,\end{aligned}$$

which does not close as long as $S > 4\|h\|$ and $\|h\| = \mathcal{O}(1)$. In the case of the 1D spinless chain (C1), we have that $\|h\| = \frac{t}{2}\cos\left(\frac{\pi}{n+1}\right)$, and so the size of the gap

$$\Delta_0 \geq 2e^{-2\sqrt{1+4\beta^2t^2}} w(2t/S)^2 \cosh(\beta t) \tag{C3}$$

scales better with β than the Gaussian filter function.

Supplementary Figure 5 shows the gap in the same setup as Supp. Fig. 4, but using a Metropolis-type filter instead of the Gaussian filter function. Qualitatively, Supp. Fig. 5 has similar features to Supp. Fig. 4, and much of the comments from above still hold. However, notice the relative difference of Δ between the $\beta = 1$ and $\beta = 3$ plots. With a Gaussian filter function, already at $\beta = 3$, the magnitude of the gap shrinks quite considerably versus the $\beta = 1$ case. The Metropolis-type filter causes similar behaviour, but to a much lesser degree, as could be expected from the analysis of Δ_0.

c. Pauli jump operators and Metropolis filter function The last modification we look at is to use different jump operators. So far, we described all operators in terms of fermionic creation and annihilation operators, and the results are therefore independent of how the mapping to qubits is performed. In this part, however, we will first map the fermionic system onto qubit operators using the Jordan-Wigner (JW) transformation (see Eq. (A10)). Performing the JW transformation manually for the spinless 1D Fermi-Hubbard model, its Hamiltonian becomes

$$\begin{aligned}H &= -t\sum_{i=1}^{n-1}(c_i^\dagger c_{i+1} + c_{i+1}^\dagger c_i) + U\sum_{i=1}^{n-1} N_i N_{i+1} \\ &\equiv \sum_{i=1}^{n-1} -\frac{t}{2}(X_iX_{i+1} + Y_iY_{i+1}) + \frac{U}{4}(Z_iZ_{i+1} + Z_{i+1} + Z_i + I).\end{aligned}$$

In this transformed system, we then use single-site Pauli operators as the jump operators in the Gibbs state preparation algorithm. The filter function remains of Metropolis-type as in the previous paragraph. Supplementary Figure 6 shows the dependence of the Lindbladian gap on the system size. While this setup is not supported by any of the theorems presented earlier in this work, it yields some interesting behaviour that is worth commenting on. We thus continue with some observations and speculative remarks regarding these results:

- *Size convergence of spinless model.* At the higher temperature of $\beta = 1$, the spinless variant of the model yields very smooth curves that — as in previous setups — show a gap that shrinks when increasing the system size. This behaviour, though, seems to approach some saturation relatively quickly, comparable to the setup with Majorana jump operators. Importantly, a key distinction is that while for the setup with Majorana operators we can prove that the gap for $U = 0$ is lower bounded for system sizes $n_{\text{sites}} \to \infty$, the same cannot be said for the setup with Pauli operators. However, the results in Supp. Fig. 6 give some confidence that the asymptotic behaviour might be similar. The lower temperature $\beta = 3$ for the spinless case shows qualitatively similar behaviour, even though the data is not quite as smooth.

- *Size convergence of spinful model.* When including spin, the data for both considered temperatures become much flatter, staying almost constant at $\beta = 1$ and seemingly fluctuating around a constant for $\beta = 3$. This could hint at even better scaling behaviour than the spinless case, even though four data points might not be enough to draw any strong conclusions.

- *Temperature stability.* A quite significant difference when using Pauli operators is the temperature dependence of the gap. Recall that with Majorana operators, at $U = 0$ a Gaussian filter function yields $\Delta_0 \sim e^{-\beta^2}$ (Eq. (C2)), and a Metropolis-type filter gives $\Delta_0 \sim e^{-\beta}$ (Eq. (C3)). Conversely, our numerics suggest that using Pauli jump operators, the temperature dependence of the gap size is quite strongly suppressed. To illustrate this point further, Supp. Fig. 7 shows the size of the Lindbladian gap depending on the interaction strength U at different temperatures $\beta = 1, 5, 25$. At high temperatures, the dependence of Δ on U is quite smooth, and the system size has relatively little influence on it. As the system is cooled down, the overall shape remains roughly the same, but much more structure with rapid oscillations emerges. Crucially, however, the magnitude of the gap seems quite unaffected by the temperature.

- *Dependence of Δ on U.* Supplementary Figure 7 also gives some confidence in regards to the stability of the gap for the one-dimensional case across a wide range of interaction strengths $-5 \lesssim U \lesssim 10$, complementing the analytical result that holds in the region around $U \approx 0$. Notice that there is a sharp drop of the gap

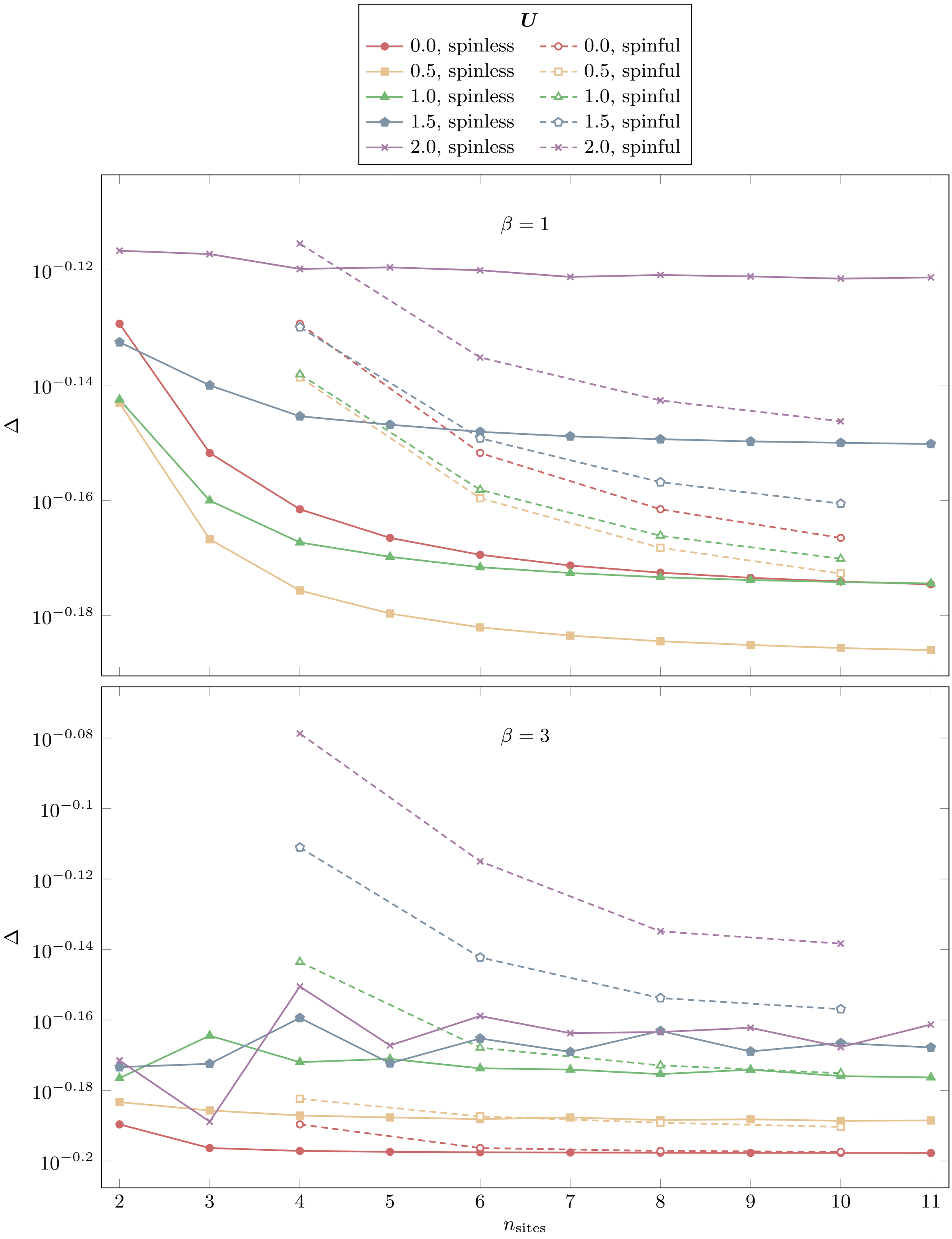

Supplementary Figure 5: Gap Δ of the Lindbladian for the spinless and spinful 1D Fermi-Hubbard model at different interaction strengths U. In the spinful model, spin-up and spin-down are counted as separate sites, resulting in only even-numbered n_{sites}. The setup is identical to that in Supplementary Figure 4, but the filter function is now of Metropolis-type, see Eq. (A8).

Δ to 0 as $|U|$ approaches the limit of the support S of the filter function (recall that $S = 10$ for these simulations). Equivalent calculations for systems in two dimensions (Supp. Fig. 8) yield similar results. Observe, however, that in this case the gap Δ closes at much smaller values of $|U|$ compared to the one-dimensional case. Still, for a given system size, there seems to be only a constant factor between the value of $|U|$ where the gap Δ is suppressed and the support size S of the filter function. This is illustrated in Supp. Fig. 9, in which the setup is identical to Supp. Fig. 8, except that S is doubled to 20. Consequently, the range of U where Δ is non-zero is also twice as large. This would suggest, for both 1D and 2D cases,

that large U can be facilitated by increasing the size of the support, which only incurs an overhead of the algorithm that is polylogarithmic in S, as per [22, Theorem 34]. Keep in mind though, that since these are only heuristic observations, some regimes of very large U might exhibit physical barriers and the gap Δ could close independently of S.

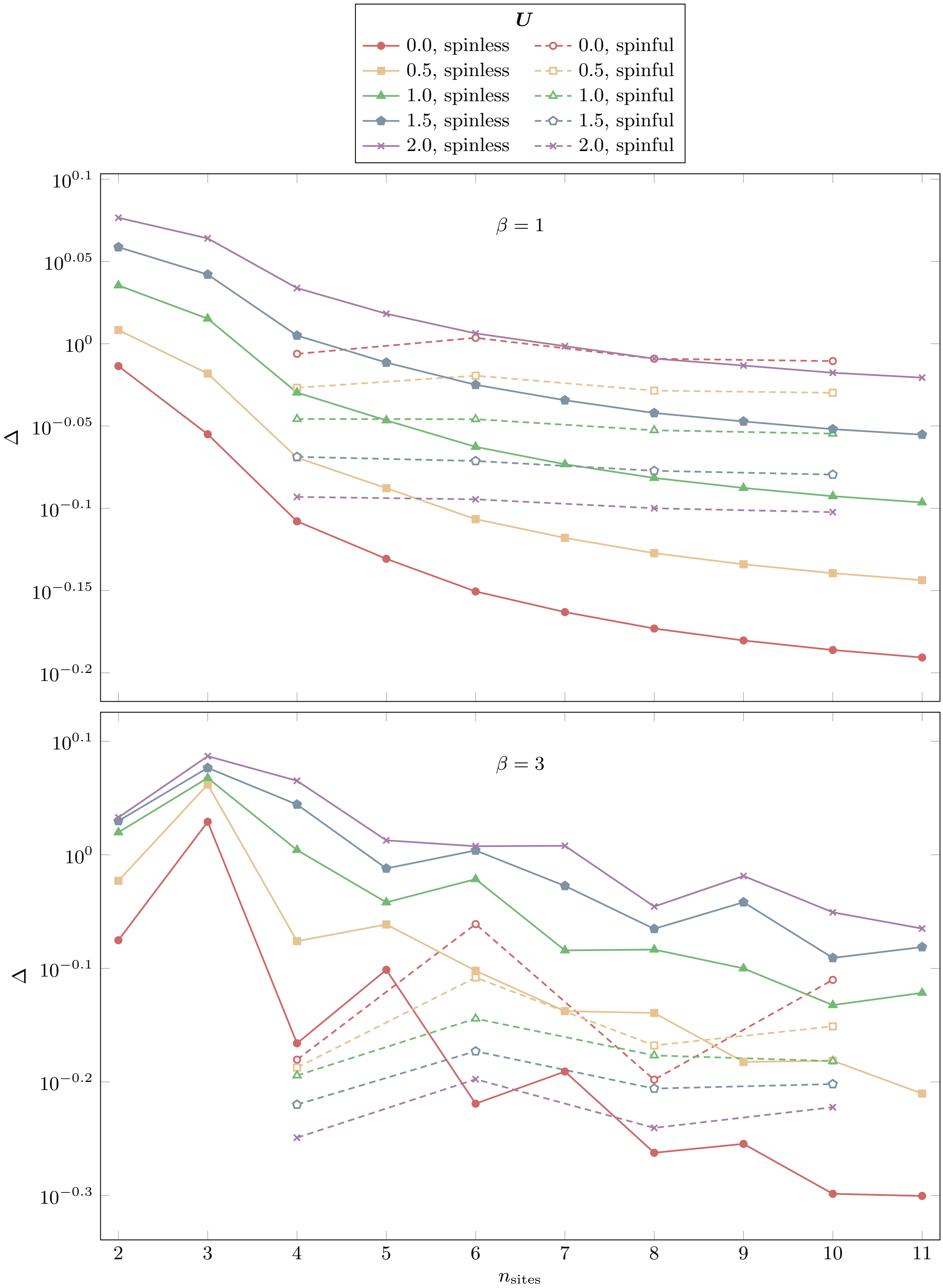

Supplementary Figure 6: Gap Δ of the Lindbladian for the spinless and spinful Fermi-Hubbard model at different interaction strengths U. In the spinful model, spin-up and spin-down are counted as separate sites, resulting in only even-numbered n_{sites}. The setup is identical to that in 5, but the jump operators are now single-site Pauli operators.

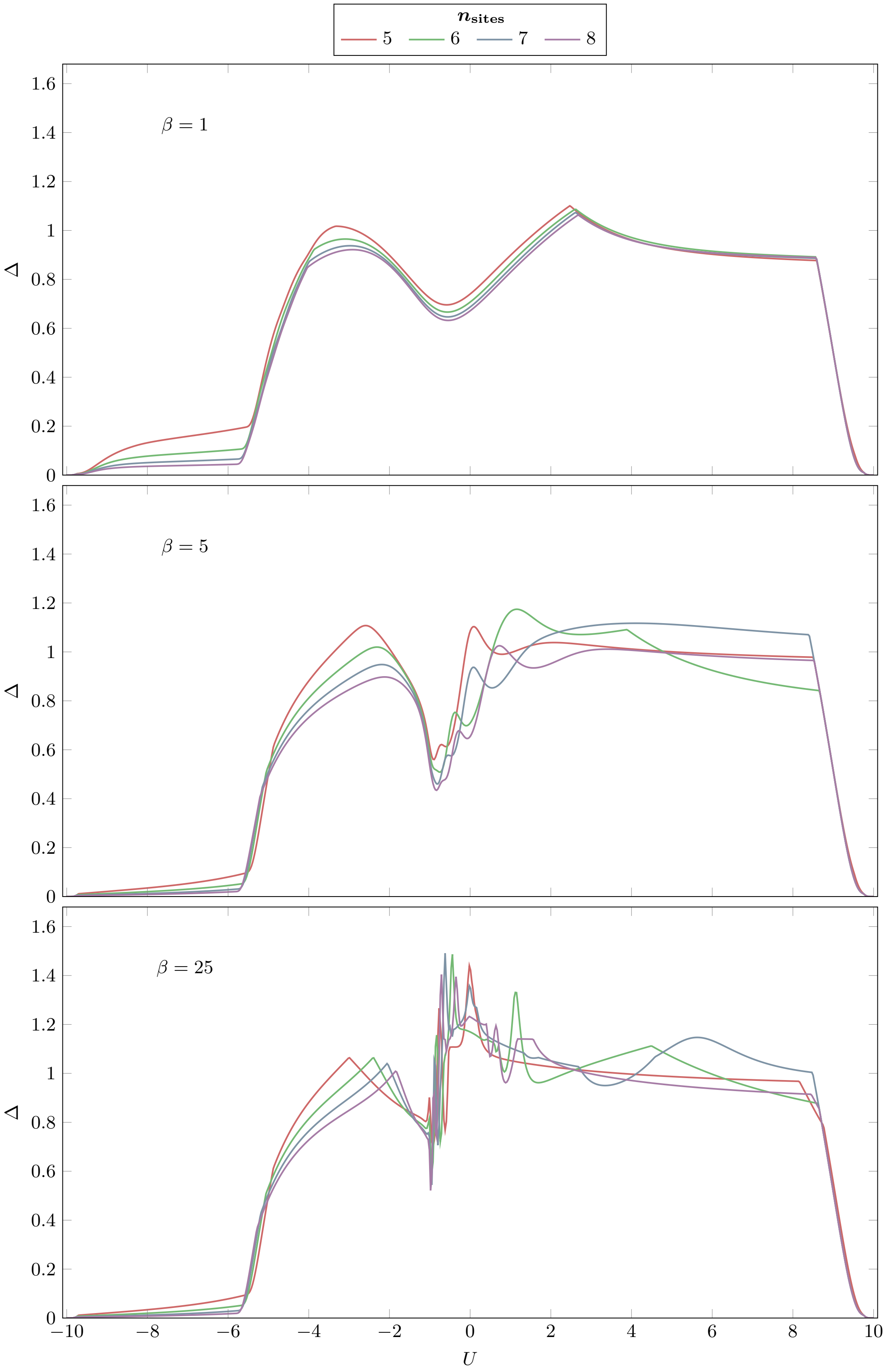

Supplementary Figure 7: Lindbladian gap Δ as a function of the interaction strength U in the spinless Fermi-Hubbard model in 1D with $t = 1$ at different inverse temperatures β when using a Metropolis-type filter function with a support of $S = 10$ and single-site Pauli operators as jump operators. Observe that the magnitude of the gap does not decay w.r.t. β, and that it closes completely for $|U| \gtrsim S$.

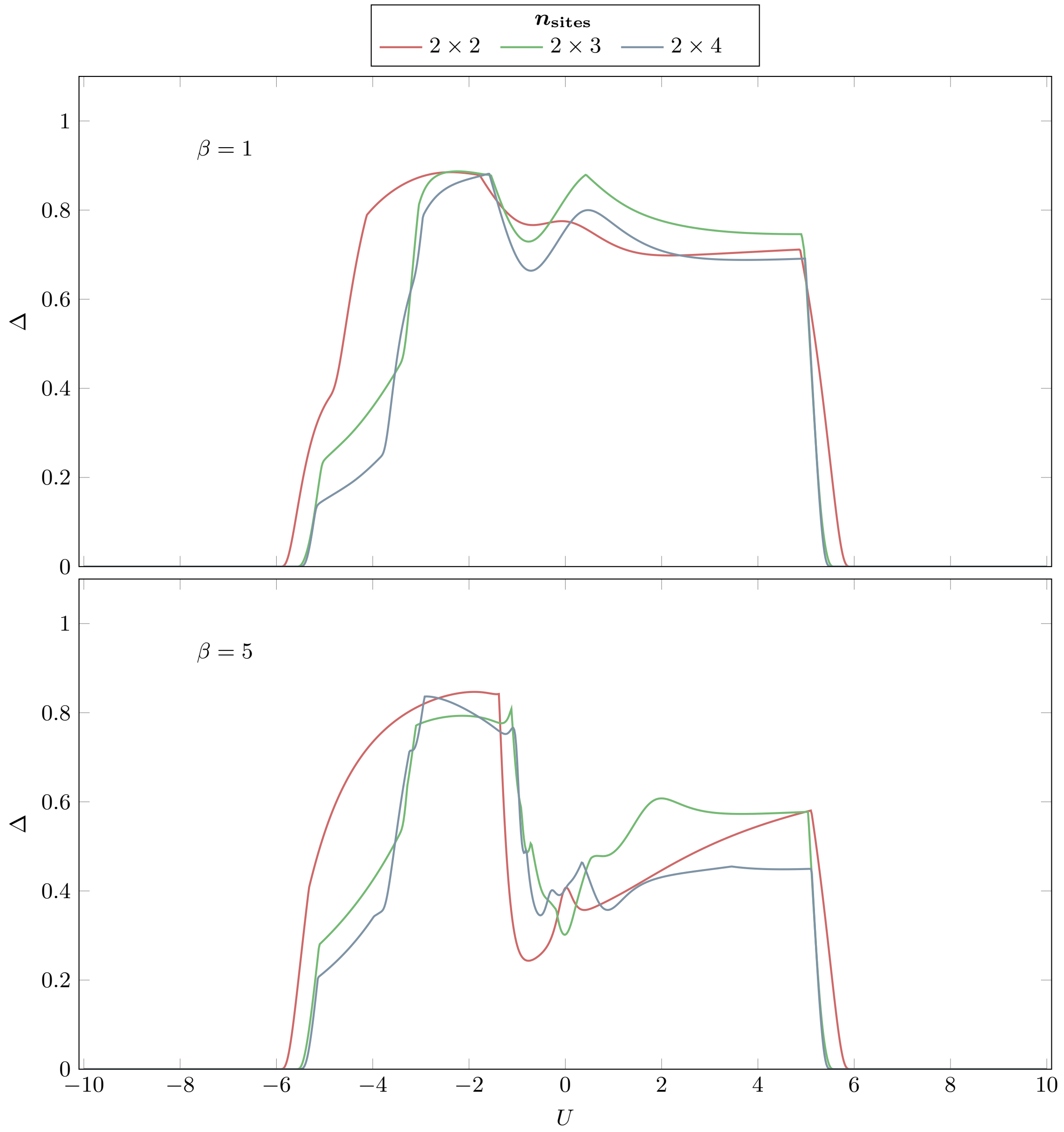

Supplementary Figure 8: Lindbladian gap Δ as a function of the interaction strength U in the spinless Fermi-Hubbard model in 2D with $t = 1$ at different inverse temperatures β when using a Metropolis-type filter function with a support of $S = 10$ and single-site Pauli operators as jump operators. Notice that compared to Supp. Fig. 7, the gap is suppressed at smaller interaction strengths $|U| < S$. However, by increasing S (see Supp. Fig. 9) we can extend the region where $\Delta > 0$ to larger $|U|$ at only polylogarithmic cost.

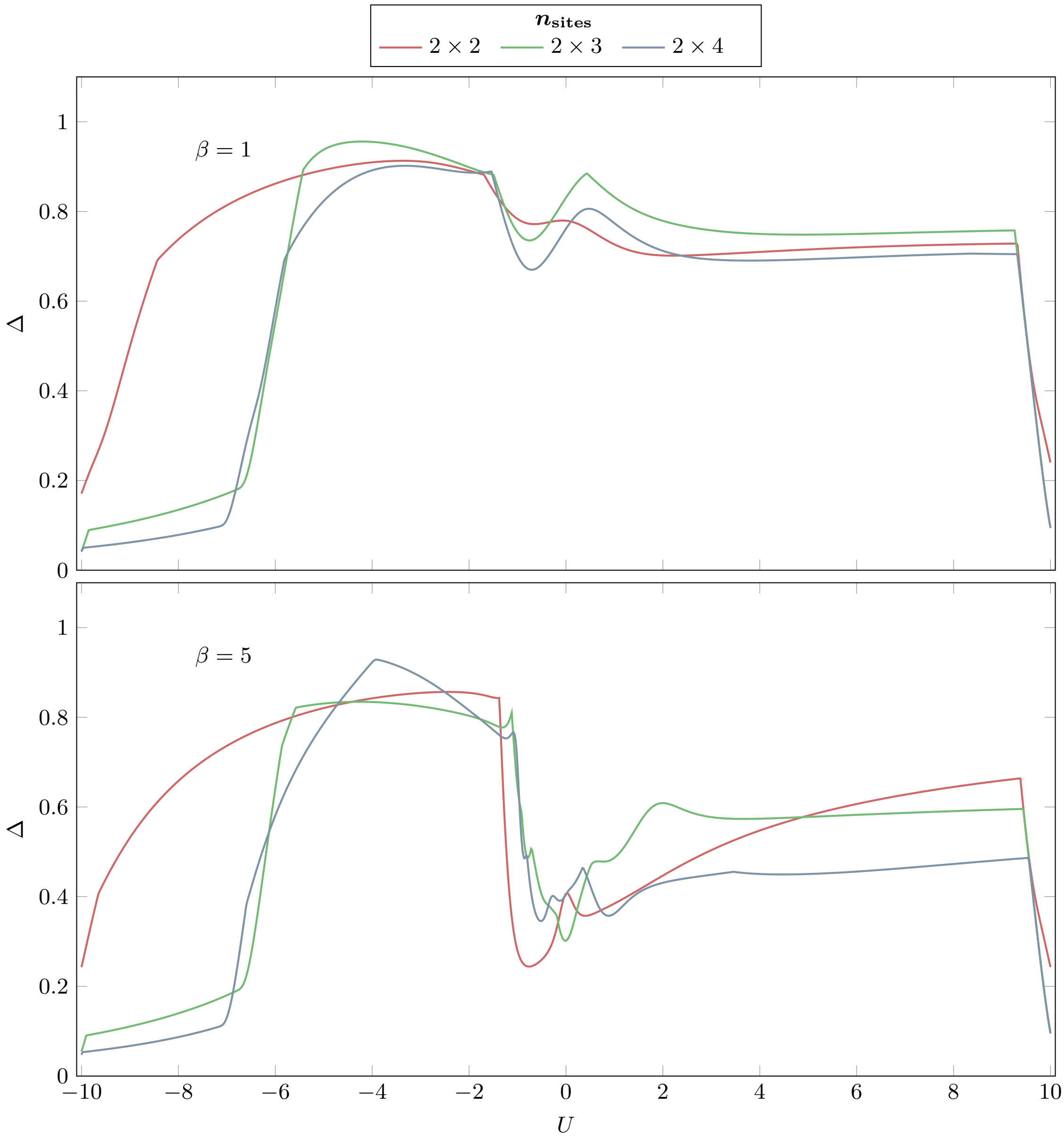

Supplementary Figure 9: Lindbladian gap Δ as a function of the interaction strength U in the spinless Fermi-Hubbard model in 2D with $t = 1$ at different inverse temperatures β when using a Metropolis-type filter function with a support of $S = 20$ and single-site Pauli operators as jump operators. Notice that because S is doubled compared to Supp. Fig. 8, the gap is supported up to twice the interaction strength $|U|$ as in Supp. Fig. 8.

Appendix D: Useful Lemmas

Lemma D.1. *For any operator O, Hermitian operators H_0, V and $\lambda, \alpha \in \mathbb{C}$, we have*

$$\|e^{\alpha(H_0+\lambda V)}Oe^{-\alpha(H_0+\lambda V)} - e^{\alpha H_0}Oe^{-\alpha H_0}\| \leq |\lambda|\,|\alpha| \max_{s\in[0,1]} \|[V, e^{s\alpha H_0}Oe^{-s\alpha H_0}]\|\,.$$

Proof. We first recall Duhamel's formula. For any operators A, B:

$$e^{(A+B)t} = e^{At} + \int_0^t e^{(A+B)(t-s)}Be^{As}\,\mathrm{d}s$$

The proof is simple: if we call $C(t)$ the right hand side, we have that

$$C'(t) = Ae^{At} + Be^{At} + \int_0^t (A+B)e^{(A+B)(t-s)}Be^{As}\,\mathrm{d}s = (A+B)C(t)\,.$$

Solving this differential equation together with $C(0) = \mathbf{1}$ yields the formula. Then denote

$$\mathcal{A} = \mathrm{ad}_{H_0}\,, \quad \mathcal{B} = \mathrm{ad}_V\,,$$

where $\mathrm{ad}_H A = [H, A]$. Let us also denote the formula we want to study by $f(\lambda, \alpha)$, so that we have

$$f(\lambda,\alpha) := \|e^{\alpha(H_0+\lambda V)} O e^{-\alpha(H_0+\lambda V)} - e^{\alpha H_0} O e^{-\alpha H_0}\| = \|(e^{\alpha(\mathcal{A}+\lambda\mathcal{B})} - e^{\alpha\mathcal{A}})O\|\,,$$

where we used the Campbell identity

$$e^{\alpha H} O e^{-\alpha H} = e^{\alpha\,\mathrm{ad}_H} O\,.$$

Duhamel's formula with $A = \alpha\mathcal{A}$, $B = \alpha\lambda\mathcal{B}$, and $t = 1$ gives:

$$f(\lambda,\alpha) = |\lambda|\,|\alpha| \left\| \int_0^1 e^{\alpha(\mathcal{A}+\lambda\mathcal{B})(1-s)} \mathcal{B} e^{\alpha\mathcal{A}s} O \,\mathrm{d}s \right\| \leq |\lambda|\,|\alpha| \int_0^1 \left\| e^{\alpha(\mathcal{A}+\lambda\mathcal{B})(1-s)} \mathcal{B} e^{\alpha\mathcal{A}s} O \right\| \,\mathrm{d}s$$

Now for any operators H and O', and $\alpha \in \mathbb{C}$, we have

$$\|e^{\alpha \mathrm{ad}_H} O'\| = \|e^{\alpha H} O' e^{-\alpha H}\| = \|O'\|\,,$$

because $e^{\alpha H} O' e^{-\alpha H}$ has the same spectrum of O'. Thus

$$f(\lambda,\alpha) \leq |\lambda|\,|\alpha| \int_0^1 \left\|\mathcal{B} e^{\alpha\mathcal{A}s} O\right\| \,\mathrm{d}s \leq |\lambda|\,|\alpha| \max_{s\in[0,1]} \left\|\mathcal{B} e^{\alpha\mathcal{A}s} O\right\|\,.$$

Plugging in the definitions of $\mathcal{A}$ and $\mathcal{B}$ gives us the result of the lemma. □

Lemma D.2. *For the Lindblad operators L_a as defined in (A3), we can express their conjugation by the Gibbs state appearing in the parent Hamiltonian as*

$$\sigma_\beta^{-1/4} L_a \sigma_\beta^{1/4} = \int_{-\infty}^{\infty} f^a(t + i\beta/4) \cdot e^{iHt} A^a e^{-iHt} \,\mathrm{d}t\,.$$

Similarly for the (individual parts of the) coherent term defined in (A4):

$$\sigma_\beta^{-1/4} G_a \sigma_\beta^{1/4} = \int_{-\infty}^{\infty} g(t + i\beta/4) \cdot e^{iHt} L_a^\dagger L_a e^{-iHt} \,\mathrm{d}t\,.$$

Proof. We need to change the contour of integration in the complex plane. For that, consider the integral

$$I = \oint_\Gamma f^a(i\beta/4 - iz) \cdot e^{Hz} A^a e^{-Hz} \,\mathrm{d}z\,,$$

where $\Gamma = \Gamma_1 + \Gamma_2 + \Gamma_T + \Gamma_B$ is the rectangular contour of integration, with $\Gamma_1 = \{z = \beta/4 + it \mid -R < t < R\}$, $\Gamma_2 = \{z = it \mid R > t > -R\}$, $\Gamma_T = \{z = iR + t \mid \beta/4 > t > 0\}$, and $\Gamma_B = \{z = -iR + t \mid 0 < t < \beta/4\}$; in the limit $R \to \infty$. As the function of interest is holomorphic, by the functional version of Cauchy's integral theorem we get that $I = 0$. We can also find that

$$\begin{aligned} I_1 &= i \cdot \sigma_\beta^{-1/4} L_a \sigma_\beta^{1/4}\,, \\ I_2 &= -i \int_{-\infty}^{\infty} f^a(t + i\beta/4) \cdot e^{iHt} A^a e^{-iHt} \,\mathrm{d}t\,, \end{aligned}$$

in the limit $R \to \infty$.

Now we want to show that the contributions I_T and I_B vanish. To do that, we can use the fact that conjugation preserves the spectral norm and that $\|A^a\| \leq 1$ to bound

$$\|I_T\| \leq \lim_{R\to\infty} \int_0^{\beta/4} |f^a(i\beta/4 - it + R)| \,\mathrm{d}t = 0\,,$$

which shows that I_T vanishes; and similarly for I_B. Hence we get that

$$\sigma_\beta^{-1/4} L_a \sigma_\beta^{1/4} = \int_{-\infty}^{\infty} f^a(t + i\beta/4) \cdot e^{iHt} A^a e^{-iHt} \,\mathrm{d}t\,.$$

The result for G follows likewise. Alternatively, to avoid any potential issues with the (unspecified) smoothened indicator function $\kappa(\nu)$ appearing in the definition of $\hat{g}(\nu)$, we present the following functional argument:

Observe that $\mathrm{ad}_H = -\frac{1}{\beta}\log(\Delta_{\sigma_\beta})$, where $\Delta_\rho[X] = \rho X \rho^{-1}$ is the modular superoperator. Hence the coherent term can be equivalently expressed as

$$G = i\tanh\left(\frac{\beta}{4}\mathrm{ad}_H\right)\left(\frac{1}{2}\sum_{a\in\mathcal{A}} L_a^\dagger L_a\right).$$

Now we can equate

$$\begin{aligned}\sigma_\beta^{-1/4} G \sigma_\beta^{1/4} = e^{\frac{\beta}{4}\mathrm{ad}_H} G = i e^{\frac{\beta}{4}\mathrm{ad}_H}\tanh\left(\frac{\beta}{4}\mathrm{ad}_H\right)\left(\frac{1}{2}\sum_{a\in\mathcal{A}} L_a^\dagger L_a\right)\\ = \hat{d}(\mathrm{ad}_H)\left(\sum_{a\in\mathcal{A}} L_a^\dagger L_a\right),\end{aligned}$$

with $\hat{d}(\nu) = \frac{i}{2}e^{\beta\nu/4}\tanh(\beta\nu/4)\kappa(\nu)$, where we've again introduced the smooth indicator function $\kappa(\nu)$, as $\kappa(\nu) = 1$ for any $\nu \in \mathrm{spec}(\mathrm{ad}_H)$. Finally we get that

$$\sigma_\beta^{-1/4} G \sigma_\beta^{1/4} = \int_{-\infty}^{\infty} g(t + i\beta/4)\cdot\sum_{a\in\mathcal{A}} e^{iHt} L_a^\dagger L_a e^{-iHt}\,\mathrm{d}t$$

by using the shifting property of the Fourier transform. □

Appendix E: Details on Bounds of Lindbladian Perturbation

The full perturbation of the parent Hamiltonian we wish to study is

$$\begin{aligned}\mathcal{V}[\rho] &= \mathcal{H}[\rho] - \mathcal{H}_0[\rho]\\ &= \sigma_\beta^{-1/4}\cdot\mathcal{L}^\dagger[\sigma_\beta^{1/4}\cdot\rho\cdot\sigma_\beta^{1/4}]\cdot\sigma_\beta^{-1/4} - \sigma_{\beta,0}^{-1/4}\cdot\mathcal{L}_0^\dagger[\sigma_{\beta,0}^{1/4}\cdot\rho\cdot\sigma_{\beta,0}^{1/4}]\cdot\sigma_{\beta,0}^{-1/4}\\ &= \sigma_\beta^{-1/4}\left(-i[G,\sigma_\beta^{1/4}\cdot\rho\cdot\sigma_\beta^{1/4}] + \sum_{a\in\mathcal{A}}\left(L_a\sigma_\beta^{1/4}\cdot\rho\cdot\sigma_\beta^{1/4}L_a^\dagger - \frac{1}{2}\{L_a^\dagger L_a, \sigma_\beta^{1/4}\cdot\rho\cdot\sigma_\beta^{1/4}\}\right)\right)\sigma_\beta^{-1/4}\\ &\quad - \sigma_{\beta,0}^{-1/4}\left(-i[G^0,\sigma_{\beta,0}^{1/4}\cdot\rho\cdot\sigma_{\beta,0}^{1/4}] + \sum_{a\in\mathcal{A}}\left(L_a^0\sigma_{\beta,0}^{1/4}\cdot\rho\cdot\sigma_{\beta,0}^{1/4}L_a^{0\dagger} - \frac{1}{2}\{L_a^{0\dagger}L_a^0, \sigma_{\beta,0}^{1/4}\cdot\rho\cdot\sigma_{\beta,0}^{1/4}\}\right)\right)\sigma_{\beta,0}^{-1/4}\\ &= -i\left(\sigma_\beta^{-1/4}G\sigma_\beta^{1/4} - \sigma_{\beta,0}^{-1/4}G^0\sigma_{\beta,0}^{1/4}\right)\cdot\rho + i\rho\cdot\left(\sigma_\beta^{1/4}G\sigma_\beta^{-1/4} - \sigma_{\beta,0}^{1/4}G^0\sigma_{\beta,0}^{-1/4}\right)\\ &\quad + \sum_{a\in\mathcal{A}}\left(\sigma_\beta^{-1/4}L_a\sigma_\beta^{1/4}\cdot\rho\cdot\sigma_\beta^{1/4}L_a^\dagger\sigma_\beta^{-1/4} - \sigma_{\beta,0}^{-1/4}L_a^0\sigma_{\beta,0}^{1/4}\cdot\rho\cdot\sigma_{\beta,0}^{1/4}L_a^{0\dagger}\sigma_{\beta,0}^{-1/4}\right)\\ &\quad - \frac{1}{2}\sum_{a\in\mathcal{A}}\left(\sigma_\beta^{-1/4}L_a^\dagger L_a\sigma_\beta^{1/4}\cdot\rho + \rho\cdot\sigma_\beta^{1/4}L_a^\dagger L_a\sigma_\beta^{-1/4} - \sigma_{\beta,0}^{-1/4}L_a^{0\dagger}L_a^0\sigma_{\beta,0}^{1/4}\cdot\rho - \rho\cdot\sigma_{\beta,0}^{1/4}L_a^{0\dagger}L_a^0\sigma_{\beta,0}^{-1/4}\right),\end{aligned}$$

from which we can consider the vectorised operator version of $\mathcal{V}$, obtained via mapping $|\psi\rangle\langle\phi| \to |\psi\rangle\overline{|\phi\rangle}$ and $O[\rho] = A\rho B \to O \cong A\otimes B^T$:

$$\begin{aligned}\mathcal{V} &\cong -i\left(\sigma_\beta^{-1/4}G\sigma_\beta^{1/4} - \sigma_{\beta,0}^{-1/4}G^0\sigma_{\beta,0}^{1/4}\right)\otimes I + iI\otimes\overline{\left(\sigma_\beta^{1/4}G\sigma_\beta^{-1/4} - \sigma_{\beta,0}^{1/4}G^0\sigma_{\beta,0}^{-1/4}\right)}\\ &\quad + \sum_{a\in\mathcal{A}}\left(\sigma_\beta^{-1/4}L_a\sigma_\beta^{1/4}\otimes\overline{\sigma_\beta^{-1/4}L_a\sigma_\beta^{1/4}} - \sigma_{\beta,0}^{-1/4}L_a^0\sigma_{\beta,0}^{1/4}\otimes\overline{\sigma_{\beta,0}^{-1/4}L_a^0\sigma_{\beta,0}^{1/4}}\right)\\ &\quad - \frac{1}{2}\sum_{a\in\mathcal{A}}\left(\sigma_\beta^{-1/4}L_a^\dagger L_a\sigma_\beta^{1/4}\otimes I + I\otimes\overline{\sigma_\beta^{1/4}L_a^\dagger L_a\sigma_\beta^{-1/4}} - \sigma_{\beta,0}^{-1/4}L_a^{0\dagger}L_a^0\sigma_{\beta,0}^{1/4}\otimes I - I\otimes\overline{\sigma_{\beta,0}^{1/4}L_a^{0\dagger}L_a^0\sigma_{\beta,0}^{-1/4}}\right).\end{aligned}$$

We wish to show that $\mathcal{V}$ has $(c|\lambda|,\mu)$-decay, and we already understand the quasi-locality of this operator given Proposition 4; hence we can focus only on upper bounding its strength. We can start by considering $\|\sigma_\beta^{-1/4}L_a\sigma_\beta^{1/4} - \sigma_{\beta,0}^{-1/4}L_a^0\sigma_{\beta,0}^{1/4}\|$, and after using the definition of L_a's, we will need to upper bound the following

expression to obtain equation (B2), where we make use of Lemma D.1:

$$\begin{aligned}
\left\|\left(e^{H(\beta/4+it)}A^a e^{-H(\beta/4+it)} - e^{H_0(\beta/4+it)}A^a e^{H_0(\beta/4+it)}\right)\right\| &\leq |\lambda||\beta/4+it| \max_{s\in[0,1]} \|[V, e^{s(\beta/4+it)H_0}A^a e^{-s(\beta/4+it)H_0}]\| \\
&= |\lambda||\beta/4+it| \max_{s\in[0,1]} \|[V, e^{s(\beta/4+it)\,\mathrm{ad}_{H_0}}\omega_a]\| \\
&= |\lambda||\beta/4+it| \max_{s\in[0,1]} \left\|\left[V, \sum_i \left(e^{-4s(\beta/4+it)h}\right)_{ai}\omega_i\right]\right\| \\
&\leq |\lambda||\beta/4+it| \max_{s\in[0,1]} \left\|e^{-4s(\beta/4+it)h}\right\|_\infty \max_i \|[V,\omega_i]\| \\
&\leq |\lambda||\beta/4+it| e^{4|\beta/4+it|\cdot\|h\|_\infty} \max_i \|[V,\omega_i]\| \\
&\leq c_2|\lambda|\cdot|\beta/4+it|\cdot e^{c_3|\beta/4+it|}\,,
\end{aligned}$$

where we then first utilised the exact solution for time evolution of ω_a, then upper bounded a weighted sum by the maximal absolute sum of the weights multiplied by the maximal element, and finally used the submultiplicativity of the ℓ_∞ norm, representing the maximal absolute row sum, to obtain a system-size-independent bound due to the assumption $\|h\|_\infty = \mathcal{O}(1)$. This expression is subsequently integrated over the Gaussian filter function $f(t)$, which is convergent, and gives us that $\|\sigma_\beta^{-1/4}L_a\sigma_\beta^{1/4} - \sigma_{\beta,0}^{-1/4}L_a^0\sigma_{\beta,0}^{1/4}\| \leq c_1|\lambda|$. The bounds on the different products of L_a's then follow immediately from this one.

Then we similarly needed to bound $\|\sigma_\beta^{-1/4}G_a\sigma_\beta^{1/4} - \sigma_{\beta,0}^{-1/4}G_a^0\sigma_{\beta,0}^{1/4}\|$:

$$\begin{aligned}
\|\sigma_\beta^{-1/4}G_a\sigma_\beta^{1/4} - \sigma_{\beta,0}^{-1/4}G_a^0\sigma_{\beta,0}^{1/4}\| &= \|e^{H\beta/4}G_a e^{-H\beta/4} - e^{H_0\beta/4}G_a^0 e^{-H_0\beta/4}\| \\
&= \left\|\int_{-\infty}^{\infty} g(t)\cdot\left(e^{H(\beta/4+it)}L_a^\dagger L_a e^{-H(\beta/4+it)} - e^{H_0(\beta/4+it)}L_a^{0\dagger}L_a^0 e^{-H_0(\beta/4+it)}\right)\,\mathrm{d}t\right\| \\
&= \left\|\int_{-\infty}^{\infty} g(t)\cdot\left(e^{H(\beta/4+it)}L_a^\dagger L_a e^{-H(\beta/4+it)} - e^{H(\beta/4+it)}L_a^{0\dagger}L_a^0 e^{-H(\beta/4+it)}\right.\right. \\
&\qquad \left.\left. + e^{H(\beta/4+it)}L_a^{0\dagger}L_a^0 e^{-H(\beta/4+it)} - e^{H_0(\beta/4+it)}L_a^{0\dagger}L_a^0 e^{-H_0(\beta/4+it)}\right)\,\mathrm{d}t\right\| \\
&\leq \int_{-\infty}^{\infty} |g(t)|\left(\left\|L_a^\dagger L_a - L_a^{0\dagger}L_a^0\right\| + \left\|e^{H(\beta/4+it)}L_a^{0\dagger}L_a^0 e^{-H(\beta/4+it)} - e^{H_0(\beta/4+it)}L_a^{0\dagger}L_a^0 e^{-H_0(\beta/4+it)}\right\|\right)\mathrm{d}t\,.
\end{aligned}$$

Here we will utilise the exact solution $L_a^0 = \sum_i \hat{f}(-4h)_{ai}\omega_i$ to analogously proceed with the following upper bound:

$$\begin{aligned}
\max_{s\in[0,1]} \|[V, e^{s(\beta/4+it)H_0}L_a^{0\dagger}L_a^0 e^{-s(\beta/4+it)H_0}]\| &\leq \|\hat{f}(-4h)\|_\infty^2 \max_{i,j}\max_{s\in[0,1]} \left\|\left[V, e^{s(\beta/4+it)H_0}\omega_i\omega_j e^{-s(\beta/4+it)H_0}\right]\right\| \\
&\leq 2\|\hat{f}(-4h)\|_\infty^2 \max_i \max_{s\in[0,1]} \left\|\left[V, e^{s(\beta/4+it)H_0}\omega_i e^{-s(\beta/4+it)H_0}\right]\right\| \\
&= 2\|\hat{f}(-4h)\|_\infty^2 \max_i \max_{s\in[0,1]} \left\|\sum_k \left(e^{-4s(\beta/4+it)h}\right)_{ik}[V,\omega_k]\right\| \\
&\leq 2\|\hat{f}(-4h)\|_\infty^2 \max_{s\in[0,1]} \left\|e^{-s\beta h}\right\|_\infty \left\|e^{-4isth}\right\|_\infty \max_k \|[V,\omega_k]\| \\
&\leq 2\|\hat{f}(-4h)\|_\infty^2 \cdot e^{\beta\|h\|_\infty}\cdot w_h(t)\cdot\max_k \|[V,\omega_k]\| \\
&\leq 2c_2 e^{c_3\beta/4}\cdot w_h(t)\cdot\|\hat{f}(-4h)\|_\infty^2\,,
\end{aligned}$$

which is then also system-size-independent due to submultiplicativity of the norm. Here, the $w_h(t) \geq \max_{s\in[0,1]}\left\|e^{-4isth}\right\|_\infty$ represents a function independent of the system size which grows subexponentially in t. A priori, we can bound $\max_{s\in[0,1]}\left\|e^{-4isth}\right\|_\infty \leq e^{4|t|\|h\|_\infty}$, which is system size independent due to $\|h\|_\infty = \mathcal{O}(1)$; but it can cause convergence issues in the integral weighted by $g(t)$. However, by Gelfand's spectral radius formula, we get that $1 = \lim_{t\to\infty}\|e^{-4isth}\|_\infty^{1/t}$ due to the orthogonality, which then implies that the growth has to be subexponential, as otherwise the limit would have to be greater than 1. One can find that for example in the 1D spinless case we consider in Section C, the norm grows like $\|e^{ith}\|_\infty \sim \sqrt{\pi t}$, and so these bounds are actually quite loose for the sparse systems we consider. Hence integrating this expression over $g(t)$ is also convergent, and we arrive at $\|\sigma_\beta^{-1/4}G_a\sigma_\beta^{1/4} - \sigma_{\beta,0}^{-1/4}G_a^0\sigma_{\beta,0}^{1/4}\| \leq c_6|\lambda|$.

While these results depend on the specifics of the Hamiltonian considered, we may state the following result, which only requires exponentially decaying correlations in the underlying system:

Lemma E.1 (General bound on decay of the perturbation). *For a Hamiltonian $H = H_0 + \lambda V$ with exponentially decaying correlations (as per the assumptions of Lieb-Robinson bounds discussed in Section A 4), there exists a constant λ_{bound}, such that for any $|\lambda| \leq \lambda_{\text{bound}}$ the perturbation of the parent Hamiltonian corresponding to the Lindbladian has (K,μ)-decay, where $K \leq c|\lambda|^{\alpha}$ for an arbitrary positive constant $\alpha < 1$, with constants c and μ being independent of the system size and λ.*

Proof. Define the truncated versions of the operators appearing in the parent Hamiltonian,

$$\begin{aligned}
\tilde{L}_a^{(r)} &= \int_{-\infty}^{\infty} f^a(t+i\beta/4) e^{iH_{B_r(a)}t} A^a e^{-iH_{B_r(a)}t}\,\mathrm{d}t\,, \\
L_a^{(r)} &= \int_{-\infty}^{\infty} f^a(t) e^{iH_{B_r(a)}t} A^a e^{-iH_{B_r(a)}t}\,\mathrm{d}t\,, \\
\tilde{G}_a^{(r)} &= \int_{-\infty}^{\infty} g(t+i\beta/4) e^{iH_{B_r(a)}t} L_a^{(r)\dagger} L_a^{(r)} e^{-iH_{B_r(a)}t}\,\mathrm{d}t\,,
\end{aligned}$$

which are supported only on the balls centred at a with radius r; and similarly $\tilde{L}_a^{0(r)}$, $L_a^{0(r)}$, and $\tilde{G}_a^{0(r)}$ for the versions corresponding to the unperturbed Hamiltonian H_0. The quasi-locality proved in Proposition 4 shows immediately that

$$\|\tilde{L}_a^{(r+1)} - \tilde{L}_a^{(r)}\| \leq c_1 e^{-\mu_1 r}\,,$$

where c_1 and μ_1 are independent of the system size. Note that these generally depend on the coupling λ, but due to their independence of system size, they must be continuous and finite for any finite λ, and so we can say that there exists some λ_{bound} below which these bounds hold for constants c_1 and μ_1 which are also independent of λ. The same bound then also holds for $\lambda = 0$ for the case $\|\tilde{L}_a^{0(r+1)} - \tilde{L}_a^{0(r)}\|$.

Now consider bounding $\|\tilde{L}_a^{(r)} - \tilde{L}_a^{0(r)}\|$:

$$\begin{aligned}
\|\tilde{L}_a^{(r)} - \tilde{L}_a^{0(r)}\| &= \left\| \int_{-\infty}^{\infty} f^a(t+i\beta/4)\left(e^{iH_{B_r(a)}t} A^a e^{-iH_{B_r(a)}t} - e^{iH^0_{B_r(a)}t} A^a e^{-iH^0_{B_r(a)}t}\right) \mathrm{d}t \right\| \\
&\leq \int_{-\infty}^{\infty} |f^a(t+i\beta/4)| \left\| e^{iH_{B_r(a)}t} A^a e^{-iH_{B_r(a)}t} - e^{iH^0_{B_r(a)}t} A^a e^{-iH^0_{B_r(a)}t}\right\| \,\mathrm{d}t \\
&\leq \int_{-\infty}^{\infty} |f^a(t+i\beta/4)||\lambda||t| \max_{s\in[0,1]} \left\| [V_{B_r(a)}, e^{istH^0_{B_r(a)}} A^a e^{-istH^0_{B_r(a)}}] \right\| \\
&\leq \int_{-\infty}^{\infty} |f^a(t+i\beta/4)| \cdot |\lambda||t| \cdot 2\|V_{B_r(a)}\| \\
&\leq c_2 r^D |\lambda|\,,
\end{aligned}$$

where we've used Lemma D.1, $\|A^a\| \leq 1$ and $\|V_{B_r(a)}\| = \mathcal{O}(r^D)$. Hence it follows that

$$\begin{aligned}
\|\tilde{L}_a^{(r+1)} \otimes \tilde{L}_a^{(r+1)} - \tilde{L}_a^{(r)} \otimes \tilde{L}_a^{(r)}\| &\leq 2c_1 e^{-\mu_1 r}\,, \\
\|\tilde{L}_a^{0(r+1)} \otimes \tilde{L}_a^{0(r+1)} - \tilde{L}_a^{0(r)} \otimes \tilde{L}_a^{0(r)}\| &\leq 2c_1 e^{-\mu_1 r}\,, \\
\|\tilde{L}_a^{(r)} \otimes \tilde{L}_a^{(r)} - \tilde{L}_a^{0(r)} \otimes \tilde{L}_a^{0(r)}\| &\leq 2c_2 r^D |\lambda|\,, \\
\|\tilde{L}_a^{(r+1)} \otimes \tilde{L}_a^{(r+1)} - \tilde{L}_a^{0(r+1)} \otimes \tilde{L}_a^{0(r+1)}\| &\leq 2c_2 (r+1)^D |\lambda| \leq 2^{D+1} c_2 r^D |\lambda|\,.
\end{aligned}$$

We can then combine these bounds to say

$$\begin{aligned}
\|\tilde{L}_a^{(r+1)} \otimes \tilde{L}_a^{(r+1)} - \tilde{L}_a^{0(r+1)} \otimes \tilde{L}_a^{0(r+1)} - \tilde{L}_a^{(r)} \otimes \tilde{L}_a^{(r)} + \tilde{L}_a^{0(r)} \otimes \tilde{L}_a^{0(r)}\| &\leq 4c_1 e^{-\mu_1 r}\,, \\
\|\tilde{L}_a^{(r+1)} \otimes \tilde{L}_a^{(r+1)} - \tilde{L}_a^{0(r+1)} \otimes \tilde{L}_a^{0(r+1)} - \tilde{L}_a^{(r)} \otimes \tilde{L}_a^{(r)} + \tilde{L}_a^{0(r)} \otimes \tilde{L}_a^{0(r)}\| &\leq (2 + 2^{D+1}) c_2 r^D |\lambda|\,.
\end{aligned}$$

Finally, as we obtained two different bounds for the same operator, we can unify them by taking their weighted geometric mean to get

$$\begin{aligned}
\|\tilde{L}_a^{(r+1)} \otimes \tilde{L}_a^{(r+1)} - \tilde{L}_a^{0(r+1)} \otimes \tilde{L}_a^{0(r+1)} - \tilde{L}_a^{(r)} \otimes \tilde{L}_a^{(r)} + \tilde{L}_a^{0(r)} \otimes \tilde{L}_a^{0(r)}\| &\leq (4c_1 e^{-\mu_1 r})^{1-\alpha} \left((2+2^{D+1}) c_2 r^D |\lambda|\right)^{\alpha} \\
&\leq c_3(\alpha) |\lambda|^{\alpha} e^{-\mu_2(\alpha) r}\,,
\end{aligned}$$

for an arbitrary positive constant $\alpha < 1$. Denoting this operator by $\epsilon_a^{1(r+1)}$, hence writing

$$\mathcal{V}^1 = \sum_{a\in\mathcal{A}} \left(\mathcal{V}_a^{1(0)} + \sum_{r\geq 1} \mathcal{V}_a^{1(r+1)} - \mathcal{V}_a^{1(r)} \right) = \sum_{a\in\mathcal{A}} \sum_{r\geq 0} \epsilon_a^{1(r)}\,,$$

we see that the first part of the perturbation $\mathcal{V}$ obeys the result of the lemma (where we made a shortcut by writing $\epsilon_a^{1(0)} = \tilde{L}_a^{(0)} \otimes \tilde{L}_a^{(0)} - \tilde{L}_a^{0(0)} \otimes \tilde{L}_a^{0(0)}$); here we have separated the perturbation into three parts like

$$\mathcal{V}^1 = \sum_{a\in\mathcal{A}} \left(\sigma_\beta^{-1/4} L_a \sigma_\beta^{1/4} \otimes \overline{\sigma_\beta^{-1/4} L_a \sigma_\beta^{1/4}} - \sigma_{\beta,0}^{-1/4} L_a^0 \sigma_{\beta,0}^{1/4} \otimes \overline{\sigma_{\beta,0}^{-1/4} L_a^0 \sigma_{\beta,0}^{1/4}}\right)$$

$$\mathcal{V}^2 = -\frac{1}{2}\sum_{a\in\mathcal{A}} \left(\sigma_\beta^{-1/4} L_a^\dagger L_a \sigma_\beta^{1/4} \otimes I + I \otimes \overline{\sigma_\beta^{1/4} L_a^\dagger L_a \sigma_\beta^{-1/4}} - \sigma_{\beta,0}^{-1/4} L_a^{0\dagger} L_a^0 \sigma_{\beta,0}^{1/4} \otimes I - I \otimes \overline{\sigma_{\beta,0}^{1/4} L_a^{0\dagger} L_a^0 \sigma_{\beta,0}^{-1/4}}\right)$$

$$\mathcal{V}^3 = -i\left(\sigma_\beta^{-1/4} G \sigma_\beta^{1/4} - \sigma_{\beta,0}^{-1/4} G^0 \sigma_{\beta,0}^{1/4}\right) \otimes I + iI \otimes \overline{\left(\sigma_\beta^{1/4} G \sigma_\beta^{-1/4} - \sigma_{\beta,0}^{1/4} G^0 \sigma_{\beta,0}^{-1/4}\right)}\,.$$

For the second part, first note that the bound $\|\tilde{L}_a^{(r)} - \tilde{L}_a^{0(r)}\|$ will also work if we conjugate $L_a^{(r)}$ by the Gibbs state with opposite exponents, amounting to changing $+i\beta/4$ to $-i\beta/4$ within the filter function in the integral. We may denote these two directions of conjugation by $\tilde{L}_a^{(r,+)}$ and $\tilde{L}_a^{(r,-)}$ respectively. Hence we arrive at

$$\|\tilde{L}_a^{(r,-)\dagger}\tilde{L}_a^{(r,+)} - \tilde{L}_a^{0(r,-)\dagger}\tilde{L}_a^{0(r,+)}\| \le c_4 r^D |\lambda|\,,$$
$$\|\tilde{L}_a^{(r+1,-)\dagger}\tilde{L}_a^{(r+1,+)} - \tilde{L}_a^{0(r+1,-)\dagger}\tilde{L}_a^{0(r+1,+)}\| \le 2c_4 r^D |\lambda|\,,$$

and the quasi-locality also gives us

$$\|\tilde{L}_a^{0(r+1,-)\dagger}\tilde{L}_a^{0(r+1,+)} - \tilde{L}_a^{0(r,-)\dagger}\tilde{L}_a^{0(r,+)}\| \le c_5 e^{-\mu_3 r}\,,$$
$$\|\tilde{L}_a^{(r+1,-)\dagger}\tilde{L}_a^{(r+1,+)} - \tilde{L}_a^{(r,-)\dagger}\tilde{L}_a^{(r,+)}\| \le c_5 e^{-\mu_3 r}\,,$$

which finally leads to

$$\|\tilde{L}_a^{(r+1,-)\dagger}\tilde{L}_a^{(r+1,+)} - \tilde{L}_a^{0(r+1,-)\dagger}\tilde{L}_a^{0(r+1,+)} - \tilde{L}_a^{(r,-)\dagger}\tilde{L}_a^{(r,+)} + \tilde{L}_a^{0(r,-)\dagger}\tilde{L}_a^{0(r,+)}\| \le c_6(\alpha)|\lambda|^\alpha e^{-\mu_4(\alpha) r}\,,$$

by the same argument as previously. This then shows that the second part of the perturbation $\mathcal{V}$ obeys the result of the lemma.

Lastly, we look at the coherent part. Here we start by bounding

$$\begin{aligned}
\|\tilde{G}_a^{(r)} - \tilde{G}_a^{0(r)}\| &= \left\|\int_{-\infty}^{\infty} g(t+i\beta/4)(e^{iH_{B_r(a)}t} L_a^{(r)\dagger} L_a^{(r)} e^{-iH_{B_r(a)}t} - e^{iH^0_{B_r(a)}t} L_a^{0(r)\dagger} L_a^{0(r)} e^{-iH^0_{B_r(a)}t})\,\mathrm{d}t\right\| \\
&\le \int_{-\infty}^{\infty} |g(t+i\beta/4)| \left\|e^{iH_{B_r(a)}t} L_a^{(r)\dagger} L_a^{(r)} e^{-iH_{B_r(a)}t} - e^{iH^0_{B_r(a)}t} L_a^{0(r)\dagger} L_a^{0(r)} e^{-iH^0_{B_r(a)}t}\right\|\,\mathrm{d}t \\
&\le \int_{-\infty}^{\infty} |g(t+i\beta/4)| \Big(\|L_a^{(r)\dagger} L_a^{(r)} - L_a^{0(r)\dagger} L_a^{0(r)}\| \\
&\qquad + \left\|e^{iH_{B_r(a)}t} L_a^{0(r)\dagger} L_a^{0(r)} e^{-iH_{B_r(a)}t} - e^{iH^0_{B_r(a)}t} L_a^{0(r)\dagger} L_a^{0(r)} e^{-iH^0_{B_r(a)}t}\right\|\Big)\,\mathrm{d}t \\
&\le c_7 r^D |\lambda| + \int_{-\infty}^{\infty} |g(t+i\beta/4)||t||\lambda| \max_{s\in[0,1]} \left\|\left[V_{B_r(a)}, e^{iH^0_{B_r(a)}st} L_a^{0(r)\dagger} L_a^{0(r)} e^{-iH^0_{B_r(a)}st}\right]\right\|\mathrm{d}t \\
&\le c_8 r^D |\lambda|\,,
\end{aligned}$$

where we've used that $\|L_a\| \le 1$ due to the normalisation of $f^a(t)$, the fact that the previous bound on $\|\tilde{L}_a^{0(r+1,-)\dagger}\tilde{L}_a^{0(r+1,+)} - \tilde{L}_a^{0(r,-)\dagger}\tilde{L}_a^{0(r,+)}\|$ also immediately works without the conjugation, and again that $\|V_{B_r(a)}\| = \mathcal{O}(r^D)$. Together with the quasi-locality of $\tilde{G}_a^{(r)}$, the same argument as before shows

$$\|\tilde{G}_a^{(r+1)} - \tilde{G}_a^{0(r+1)} - \tilde{G}_a^{(r)} - \tilde{G}_a^{0(r)}\| \le c_9(\alpha)|\lambda|^\alpha e^{-\mu_5(\alpha) r}\,,$$

which leads to the third and final part of $\mathcal{V}$ to obey the result of the lemma, meaning it holds for the full $\mathcal{V}$, as

$$\mathcal{V} = \sum_{a\in\mathcal{A}} \sum_{t=1}^{3} \sum_{r\ge 0} \epsilon_a^{t(r)},$$

where $\|\epsilon_a^{t(r)}\| \le c(\alpha)|\lambda|^\alpha e^{-\mu(\alpha) r}$; hence finishing the proof. □

[1] Dalzell, A. M. *et al.* *Quantum Algorithms: A Survey of Applications and End-to-end Complexities* (Cambridge University Press, 2025).
[2] Kitaev, A. Y., Shen, A. & Vyalyi, M. N. *Classical and Quantum Computation* (American Mathematical Society, 2002).
[3] Chen, C.-F., Kastoryano, M., Brandão, F. G. S. L. & Gilyén, A. Efficient quantum thermal simulation. *Nature* **646**, 561–566 (2025).
[4] Levin, D. A. & Peres, Y. *Markov Chains and Mixing Times* (American Mathematical Society, 2017).
[5] Aharonov, D., Gottesman, D., Irani, S. & Kempe, J. The power of quantum systems on a line. *Communications in Mathematical Physics* **287**, 41–65 (2009).
[6] Schuch, N. & Verstraete, F. Computational complexity of interacting electrons and fundamental limitations of density functional theory. *Nature physics* **5**, 732–735 (2009).
[7] O'Gorman, B., Irani, S., Whitfield, J. & Fefferman, B. Intractability of electronic structure in a fixed basis. *PRX Quantum* **3**, 020322 (2022).
[8] Temme, K., Osborne, T. J., Vollbrecht, K. G., Poulin, D. & Verstraete, F. Quantum Metropolis sampling. *Nature* **471**, 87–90 (2011).
[9] Yung, M.-H. & Aspuru-Guzik, A. A quantum–quantum Metropolis algorithm. *Proceedings of the National Academy of Sciences* **109**, 754–759 (2012).
[10] Shtanko, O. & Movassagh, R. Preparing thermal states on noiseless and noisy programmable quantum processors (2021). 2112.14688.
[11] Moussa, J. E. Low-depth quantum Metropolis algorithm (2022). 1903.01451.
[12] Rall, P., Wang, C. & Wocjan, P. Thermal State Preparation via Rounding Promises. *Quantum* **7**, 1132 (2023).
[13] Wocjan, P. & Temme, K. Szegedy walk unitaries for quantum maps. *Communications in Mathematical Physics* **402**, 3201–3231 (2023).
[14] Jiang, J. & Irani, S. Quantum Metropolis sampling via weak measurement (2024). 2406.16023.
[15] Chowdhury, A. N. & Somma, R. D. Quantum algorithms for Gibbs sampling and hitting-time estimation. *Quantum Information & Computation* **17**, 0041–0064 (2017).
[16] van Apeldoorn, J., Gilyén, A., Gribling, S. & de Wolf, R. Quantum SDP-Solvers: Better upper and lower bounds. *Quantum* **4**, 230 (2020).
[17] van Apeldoorn, J. & Gilyén, A. Improvements in quantum SDP-Solving with applications. In *46th International Colloquium on Automata, Languages, and Programming (ICALP 2019)*, vol. 132, 99:1–99:15 (Schloss Dagstuhl – Leibniz-Zentrum für Informatik, 2019).
[18] Gilyén, A., Su, Y., Low, G. H. & Wiebe, N. Quantum singular value transformation and beyond: exponential improvements for quantum matrix arithmetics. In *Proceedings of the 51st Annual ACM SIGACT Symposium on Theory of Computing*, STOC 2019, 193–204 (Association for Computing Machinery, New York, NY, USA, 2019).
[19] An, D., Childs, A. M. & Lin, L. Quantum algorithm for linear non-unitary dynamics with near-optimal dependence on all parameters (2023). 2312.03916.
[20] Mozgunov, E. & Lidar, D. Completely positive master equation for arbitrary driving and small level spacing. *Quantum* **4**, 227 (2020).
[21] Nathan, F. & Rudner, M. S. Universal Lindblad equation for open quantum systems. *Phys. Rev. B* **102**, 115109 (2020).
[22] Ding, Z., Li, B. & Lin, L. Efficient quantum Gibbs samplers with Kubo–Martin–Schwinger detailed balance condition. *Communications in Mathematical Physics* **406**, 67 (2025).
[23] Gilyén, A., Chen, C.-F., Doriguello, J. F. & Kastoryano, M. J. Quantum generalizations of Glauber and Metropolis dynamics (2024). 2405.20322.
[24] Albash, T. & Lidar, D. A. Adiabatic quantum computation. *Rev. Mod. Phys.* **90**, 015002 (2018).
[25] Temme, K. & Kastoryano, M. J. How fast do stabilizer Hamiltonians thermalize? (2015). 1505.07811.
[26] Kastoryano, M. J. & Brandão, F. G. S. L. Quantum Gibbs samplers: The commuting case. *Communications in Mathematical Physics* **344**, 915–957 (2016).
[27] Brandão, F. G. S. L. & Kastoryano, M. J. Finite correlation length implies efficient preparation of quantum thermal states. *Communications in Mathematical Physics* **365**, 1–16 (2019).
[28] Bardet, I. *et al.* Entropy decay for Davies semigroups of a one dimensional quantum lattice. *Communications in Mathematical Physics* **405**, 42 (2024).
[29] Kochanowski, J., Alhambra, A. M., Capel, A. & Rouzé, C. Rapid thermalization of dissipative many-body dynamics of commuting Hamiltonians (2024). 2404.16780.
[30] Rouzé, C., França, D. S. & Alhambra, A. M. Efficient thermalization and universal quantum computing with quantum Gibbs samplers. In *Proceedings of the 57th Annual ACM Symposium on Theory of Computing*, STOC '25, 1488–1495 (Association for Computing Machinery, New York, NY, USA, 2025).
[31] Rouzé, C., França, D. S. & Alhambra, Á. M. Optimal quantum algorithm for Gibbs state preparation (2024). 2411.04885.
[32] Bakshi, A., Liu, A., Moitra, A. & Tang, E. High-temperature Gibbs states are unentangled and efficiently preparable (2024). 2403.16850.
[33] Ramkumar, A. & Soleimanifar, M. Mixing time of quantum Gibbs sampling for random sparse Hamiltonians (2024). 2411.04454.
[34] Basso, J., Chen, C.-F. & Dalzell, A. M. Optimizing random local Hamiltonians by dissipation (2024). 2411.02578.
[35] Ding, Z., Li, B., Lin, L. & Zhang, R. Polynomial-time preparation of low-temperature Gibbs states for 2D toric code (2024). 2410.01206.
[36] Bergamaschi, T., Chen, C.-F. & Liu, Y. Quantum computational advantage with constant-temperature Gibbs sampling (2024). 2404.14639.
[37] Rajakumar, J. & Watson, J. D. Gibbs sampling gives quantum advantage at constant temperatures with O(1)-local Hamiltonians (2024). 2408.01516.
[38] Bärtschi, A. *et al.* Potential applications of quantum computing at Los Alamos National Laboratory (2024). 2406.06625.
[39] Arovas, D. P., Berg, E., Kivelson, S. A. & Raghu, S. The Hubbard model. *Annual Review of Condensed Matter Physics* **13**, 239–274 (2022).
[40] Qin, M., Schäfer, T., Andergassen, S., Corboz, P. & Gull, E. The Hubbard model: A computational perspective. *Annual Review of Condensed Matter Physics* **13**, 275–302 (2022).
[41] Chan, G. K.-L. Quantum chemistry, classical heuristics, and quantum advantage (2024). 2407.11235.
[42] Gross, C. & Bloch, I. Quantum simulations with ultracold atoms in optical lattices. *Science* **357**, 995–1001 (2017).

[43] Anschuetz, E. R., Chen, C.-F., Kiani, B. T. & King, R. Strongly interacting fermions are nontrivial yet nonglassy. *Phys. Rev. Lett.* **135**, 030602 (2025).
[44] Prosen, T. Third quantization: a general method to solve master equations for quadratic open Fermi systems. *New Journal of Physics* **10**, 043026 (2008).
[45] Bittel, L., Mele, A. A., Eisert, J. & Leone, L. Optimal trace-distance bounds for free-fermionic states: Testing and improved tomography (2025). 2409.17953.
[46] Hastings, M. B. The stability of free Fermi Hamiltonians (2017). 1706.02270.
[47] De Roeck, W. & Salmhofer, M. Persistence of exponential decay and spectral gaps for interacting fermions. *Communications in Mathematical Physics* **365**, 773–796 (2018).
[48] Koma, T. Stability of the spectral gap for lattice fermions (2020). 2005.04548.
[49] Mann, R. L. & Helmuth, T. Efficient algorithms for approximating quantum partition functions. *Journal of Mathematical Physics* **62** (2021).
[50] Essler, F. H., Frahm, H., Göhmann, F., Klümper, A. & Korepin, V. E. *The one-dimensional Hubbard model* (Cambridge University Press, 2005).
[51] Liu, W.-Y., Zhai, H., Peng, R., Gu, Z.-C. & Chan, G. K.-L. Accurate simulation of the Hubbard model with finite fermionic projected entangled pair states (2025). 2502.13454.
[52] Helmuth, T. & Mann, R. L. Efficient algorithms for approximating quantum partition functions at low temperature. *Quantum* **7**, 1155 (2023).
[53] Šmíd, Š., Meister, R., Berta, M. & Bondesan, R. Polynomial-time quantum Gibbs sampling for the weak and strong coupling regime of the Fermi-Hubbard model at any temperature. *Quantum-Gibbs-Sampling* (2025). URL https://doi.org/10.5281/zenodo.17390410.
[54] Krzakała, F., Montanari, A., Ricci-Tersenghi, F., Semerjian, G. & Zdeborová, L. Gibbs states and the set of solutions of random constraint satisfaction problems. *Proceedings of the National Academy of Sciences* **104**, 10318–10323 (2007). https://www.pnas.org/doi/pdf/10.1073/pnas.0703685104.
[55] Somma, R. D., Boixo, S., Barnum, H. & Knill, E. Quantum simulations of classical annealing processes. *Phys. Rev. Lett.* **101**, 130504 (2008).
[56] Amin, M. H., Andriyash, E., Rolfe, J., Kulchytskyy, B. & Melko, R. Quantum boltzmann machine. *Physical Review X* **8** (2018).
[57] Biamonte, J. & Bergholm, V. Tensor networks in a nutshell (2017). 1708.00006.
[58] Mortier, Q. *et al.* Fermionic tensor network methods (2024). 2404.14611.
[59] Barthel, T. & Zhang, Y. Solving quasi-free and quadratic Lindblad master equations for open fermionic and bosonic systems. *Journal of Statistical Mechanics: Theory and Experiment* **2022**, 113101 (2022).
[60] Haah, J., Hastings, M. B., Kothari, R. & Low, G. H. Quantum algorithm for simulating real time evolution of lattice Hamiltonians. *SIAM Journal on Computing* **52**, FOCS18–250–FOCS18–284 (2021).
[61] Nachtergaele, B., Sims, R. & Young, A. Lieb-Robinson bounds, the spectral flow, and stability of the spectral gap for lattice fermion systems. *Mathematical Problems in Quantum Physics* **717**, 93–115 (2018).
[62] Tong, Y. & Zhan, Y. Fast mixing of weakly interacting fermionic systems at any temperature. *PRX Quantum* **6**, 030301 (2025).
[63] Harvey, M. Imperial College research computing service (2017).
[64] Wolf, M. M. Quantum channels & operations: Guided tour. *Niels-Bohr Institute* (2012). URL https://mediatum.ub.tum.de/doc/1701036/document.pdf/.
[65] Knill, E., Ortiz, G. & Somma, R. D. Optimal quantum measurements of expectation values of observables. *Physical Review A* **75** (2007).
[66] Avdoshkin, A. & Dymarsky, A. Euclidean operator growth and quantum chaos. *Physical Review Research* **2** (2020).
[67] Schweitzer, M. Decay bounds for Bernstein functions of Hermitian matrices with applications to the fractional graph Laplacian (2021). 2111.06135.
[68] Bravyi, S. & Hastings, M. B. A short proof of stability of topological order under local perturbations. *Communications in Mathematical Physics* **307**, 609–627 (2011).
[69] Michalakis, S. & Zwolak, J. P. Stability of frustration-free Hamiltonians. *Communications in Mathematical Physics* **322**, 277–302 (2013).
[70] Datta, N., Fernández, R. & Fröhlich, J. Low-temperature phase diagrams of quantum lattice systems. I. stability for quantum perturbations of classical systems with finitely-many ground states. *Journal of statistical physics* **84**, 455–534 (1996).
[71] Borgs, C. & Kotecký, R. Low temperature phase diagrams of fermionic lattice systems. *Communications in Mathematical Physics* **208**, 575–604 (2000).
